# Essential role of obscurin kinase-1 in cardiomyocyte coupling via N-cadherin phosphorylation

**DOI:** 10.1172/jci.insight.162178

**Published:** 2024-02-08

**Authors:** Li Wang, Panagiotis Tsakiroglou, Rex Gonzales, Suhan Cho, Amy Li, Cristobal dos Remedios, Nathan Wright, Aikaterini Kontrogianni-Konstantopoulos

**Affiliations:** 1 Department of Biochemistry and Molecular Biology, University of Maryland School of Medicine, Baltimore, Maryland, USA.; 2 Sydney Heart Bank, University of Sydney, Australia.; 3 Department of Rural Clinical Sciences, La Trobe University, Bendigo, Victoria, Australia.; 4 Centre for Healthy Futures, Torrens University Australia, Surry Hills, New South Wales, Australia.; 5 Victor Chang Cardiac Research Institute, Darlinghurst, New South Wales, Australia.; 6 Department of Chemistry and Biochemistry, James Madison University, Harrisonburg, Virginia, USA.

**Keywords:** Cell Biology, Cytoskeleton, Signal transduction

## Abstract

Obscurins are giant cytoskeletal proteins with structural and regulatory roles. Obscurin-B (~870 kDa), the largest known isoform, contains 2 enzymatically active Ser/Thr kinase (kin) domains, kin1 and kin2, which belong to the myosin light chain kinase family. Kin1 binds to and phosphorylates N-cadherin, a major component of the intercalated disc, the unique sarcolemmal microdomain that mediates the mechanochemical coupling of adjacent cardiomyocytes. Obscurin-B containing kin1 and N-cadherin colocalize at cell junctions in embryonic rat ventricular myocytes (ERVMs), and their codistribution is regulated by Ca^2+^. Phosphoproteomics analysis revealed that obscurin-kin1 phosphorylates N-cadherin at Ser-788 located within the juxtamembrane region of its cytoplasmic domain, with an apparent *K_cat_* of approximately 5.05 min^–1^. Overexpression of obscurin-kin1 or phosphomimic-Ser-788-Glu N-cadherin in ERVMs markedly increases cell adhesion and chemical coupling. Importantly, phosphomimic Ser-788-Glu N-cadherin exhibits significantly reduced binding to p120-catenin, while overexpression of phosphoablated Ser-788-Ala N-cadherin increases RhoA activity. Consistent with an essential role of the obscurin-kin1/N-cadherin axis in cardiomyocyte coupling, it is deregulated in end-stage human heart failure. Given the nearly ubiquitous expression of obscurin and N-cadherin, our findings may have broad applicability in deciphering the obscurin-kin1/N-cadherin axis that likely mediates cell coupling in diverse tissues and organs.

## Abstract 2

<p>The obscurin kinase-1/phospho-N-cadherin axis at the cardiac intercalated disc plays a key role in cardiomyocyte coupling and is deregulated in heart failure.</p>

## Introduction

Obscurins encoded by the *OBSCN* gene comprise a family of giant cytoskeletal regulators exhibiting nearly ubiquitous expression (1), with the highest prevalence in striated muscles (2, 3) where they play key roles in the organization of thick filaments, the sarcomeric alignment of internal membranes, the maintenance of the subsarcolemmal microtubule network, the modulation of RhoA and PI3K axes, and more recently the regulation of Ca^2+^ cycling (4–18). Consistent with the diverse roles of obscurins in muscle cells, missense, nonsense, and frameshift mutations in *OBSCN* have been linked to different forms of cardiac and skeletal myopathies (19).

The prototypical obscurin, referred to as obscurin-A (~720 kDa), is composed of tandem immunoglobulin (Ig) and fibronectin type III (FnIII) domains followed by a cluster of signaling domains arranged in tandem and a nonmodular COOH terminus that contains ankyrin binding sites (3, 20, 21). Obscurin-B (~870 kDa), the largest known obscurin isoform, shares the same modular architecture with obscurin-A, with the exception of its COOH terminus that contains 2 Ser/Thr kinase domains, kin1 and kin2, that belong to the myosin light chain kinase subfamily (22, 23). Although the obscurin kinase domains have been identified for 2 decades, their enzymatic activity, regulation, substrates, and roles have remained enigmatic and at times controversial. Our group demonstrated that both kin1 and kin2 are enzymatically active, and that kin1 binds to and phosphorylates the cytoplasmic domain of N-cadherin in vitro (24).

N-cadherin is a Ca^2+^-dependent transmembrane protein that is the principal component of adherens junctions (AJ) present at the intercalated disc (ICD), the unique sarcolemmal microdomain found at the bipolar ends of cardiomyocytes that mediates their mechanochemical coupling (25, 26). N-cadherin is the only classical type I cadherin expressed in the ICD, where it has a dual role; through its extracellular portion it connects adjoining cardiomyocytes in a homophilic way, while through its cytoplasmic portion it interacts with intracellular adaptor proteins, including a set of catenins and catenin-related proteins, linking the underlying cytoskeleton to the cell membrane (25, 27). The pivotal role of N-cadherin in maintaining cardiac structure and function has been demonstrated in several studies. As such, constitutive loss of N-cadherin in early development results in embryonic lethality, due to severe cardiovascular defects (28). Conversely, cardiac-specific loss of N-cadherin during adulthood leads to disassembly of the ICD, myofibrillar disarray, and dilated cardiomyopathy (DCM) accompanied by ventricular arrhythmia and eventually sudden cardiac death (29–31).

To date, the molecular mechanisms that regulate the functional properties of N-cadherin have only been cursorily examined. Accordingly, Lee et al. demonstrated that N-cadherin is a target of endogenous kinases and phosphatases, and that its phosphorylation state is an important determinant of its stability and functionality at the cell surface (32). The same authors also reported that native N-cadherin contains high basal levels of Ser phosphorylation in the heart, retina, brain, and lens, while upon stimulation it undergoes further Ser and Tyr phosphorylation (32). Along these lines, Qi et al. documented that phosphorylation of N-cadherin by Src kinase on Tyr-860 abolishes its ability to bind β-catenin at the plasma membrane of melanoma cells (33). Thus, phosphorylation of the cytoplasmic domain of N-cadherin is a potent mechanism that may regulate its stability, targeting, binding interactions, and mechanical/adhesive properties.

Herein, we expand the roles of *OBSCN* in cell adhesion by showing that obscurin-kin1 is essential in the mechanochemical coupling of cardiomyocytes via phosphorylation of the cytoplasmic domain of N-cadherin at Ser-788. Our studies are of high pathophysiological relevance, as they unravel what we believe is a previously unknown molecular mechanism contributing to the regulation of cardiomyocyte adhesion and communication, which is deregulated in heart failure. Given the nearly ubiquitous expression of obscurins and N-cadherin, our findings may have broad applicability in characterizing a regulatory axis that likely mediates cell coupling in diverse tissues and organs.

## Results

### Ca^2+^-regulated distribution of obscurin-kin1 and N-cadherin at cell junctions.

**Figure 1:**
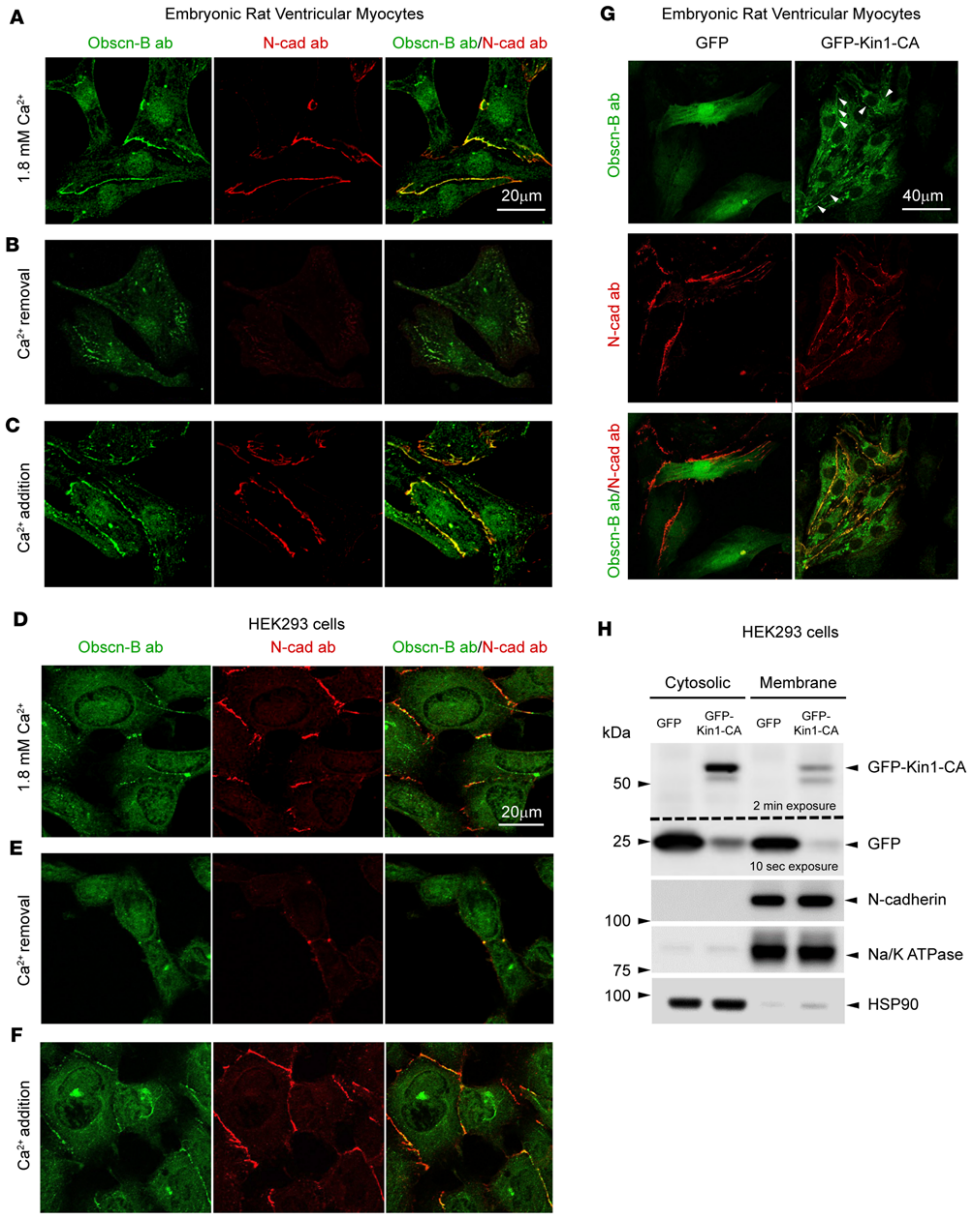
The codistribution of obscurin-B containing kin1 and N-cadherin at cell junctions is coordinately regulated by Ca^2+^. (**A**–**F**) Ca^2+^ switch experiments combined with confocal imaging demonstrated that obscurin-B colocalizes with N-cadherin at cell junctions in primary cultures of ERVMs (**A**) and HEK293 cells (**D**) in the presence of Ca^2+^. Ca^2+^ removal for 4 hours resulted in loss of both obscurin-B and N-cadherin from cell junctions, with residual proteins exhibiting a punctate or diffuse cytoplasmic distribution in ERVMs (**B**) and HEK293 cells (**E**), respectively. Addition of Ca^2+^ in the medium for 1 hour restored the codistribution of obscurin-B and N-cadherin at cell junctions in both ERVMs (**C**) and HEK293 cells (**F**); *n* = 4 experiments for ERVM (4 optical fields per condition, per experiment) and *n* = 2 for HEK293 cells (5–6 optical fields per condition, per experiment). (**G**) Confocal evaluation of ERVM cultures transfected with control GFP and GFP-Kin1-CA indicated that exogenous GFP-Kin1-CA, but not GFP protein, colocalizes with endogenous N-cadherin at cell contact sites (arrowheads); *n* = 3 experiments. (**H**) Cell fractionation of HEK293 cells transfected with control GFP or GFP-Kin1-CA followed by immunoblotting analysis showed that approximately 36% of exogenous GFP-Kin1-CA is found in the membrane fraction, similarly to endogenous N-cadherin; the identity of the membrane and cytoplasmic fractions was confirmed by probing for Na/K ATPase and HSP90, respectively, which also served as loading controls within each fraction; *n* = 3 experiments. Scale bars: 20 μm (**A** and **D**–**F**) and 40 μm (**G**).

Prior work from our group demonstrated that obscurin-kin1 specifically binds to and phosphorylates the cytoplasmic domain of N-cadherin in vitro (24). This finding was further supported by the coincident distribution of obscurin-B containing kin1 and N-cadherin at cell junctions in the ICD of adult cardiomyocytes (24). Given that the membrane targeting of N-cadherin is Ca^2+^ dependent (34), we performed Ca^2+^ switch experiments to examine whether the preferential distribution of obscurin-B containing kin1 and N-cadherin at cell junctions is coordinately regulated by Ca^2+^. We found that endogenous obscurin-B containing kin1 and N-cadherin codistributed at cell junctions in both embryonic rat ventricular myocytes (ERVMs) (Figure 1A) and HEK293 cells (Figure 1D), when grown in the presence of standard culture medium containing 1.8 mM CaCl_2_. Removal of Ca^2+^ for 4 hours resulted in loss of both obscurin-B and N-cadherin from cell junctions, with residual proteins assuming a punctate and diffuse distribution in ERVMs (Figure 1B) and HEK293 cells (Figure 1E), respectively. Importantly, addition of 1.8 mM CaCl_2_ in the culture medium resulted in redistribution of obscurin-B and N-cadherin to cell junctions in both ERVMs (Figure 1C) and HEK293 cells (Figure 1F) within 1 hour.

Similar to endogenous obscurin-B, ectopically expressed GFP-tagged obscurin-kin1 containing the catalytic domain (GFP-Kin1-CA), but not control GFP protein, targeted to the cell membrane in ERVMs, with a preferential accumulation at cell junctions where it colocalized with endogenous N-cadherin (Figure 1G), consistent with their direct association (24). In addition to its membrane localization, GFP-Kin1-CA also exhibited a punctate distribution in the cytoplasm. The codistribution of exogenous GFP-Kin1-CA and endogenous N-cadherin at the cell membrane was further confirmed by subcellular fractionation of HEK293 cells where approximately 36% of exogenous GFP-Kin1-CA was found in the membrane fraction, where endogenous N-cadherin was also detected (Figure 1H). Taken together, these findings support the coordinated, Ca^2+^-dependent codistribution of obscurin-B containing kin1 and N-cadherin at cell junctions, implying a (patho)physiological relevance of their association.

### Obscurin-kin1 regulates cardiomyocyte adhesion and communication.

**Figure 2:**
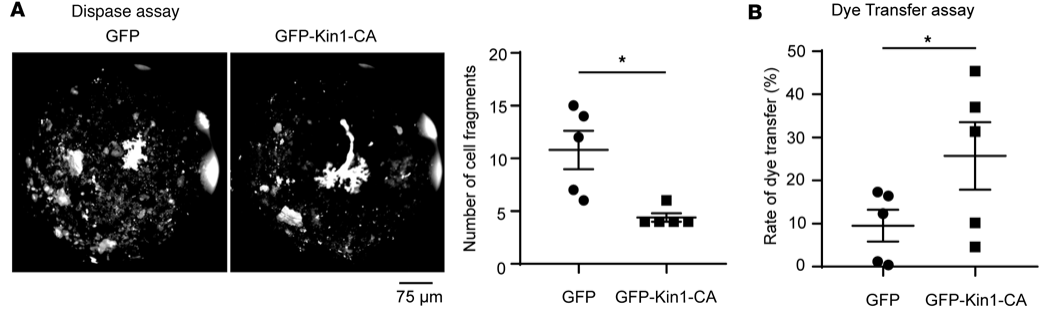
Obscurin-kin1 modulates cardiomyocyte mechanochemical coupling. Overexpression of GFP-Kin1-CA, but not control GFP protein, in ERVMs significantly increases cardiomyocyte adhesion as shown by the reduced number of cell fragments (≥100 mm) obtained in a Dispase assay (**A**; *n* = 5, two-tailed paired *t* test, *P = 0.0362), and intercellular communication as indicated by the increased rate of dye transfer (**B**; *n* = 5, two-tailed paired *t* test, *P = 0.0206). Data are expressed as mean ± SEM.

Given the association and coregulated distribution of obscurin-B containing kin1 and N-cadherin at cell junctions, we next investigated the role of kin1 in mediating cardiomyocyte adhesion and communication. To this end, we introduced control GFP and GFP-Kin1-CA in ERVM cultures and following cell sorting via flow cytometry we collected and assayed the GFP-positive cell populations. We first performed a Dispase mechanical dissociation assay to examine the impact of GFP-Kin1-CA in modulating cardiomyocyte adhesion. ERVMs expressing GFP-Kin1-CA exhibited a significantly lower number of cell fragments compared with ERVMs expressing control GFP (Figure 2A), indicating that overexpression of GFP-Kin1-CA strengthens intercellular adhesion. Next, we investigated the effect of GFP-Kin1-CA in regulating intercellular communication via a dye transfer assay. Donor ERVMs expressing GFP or GFP-Kin1-CA were labeled with the gap junction–transferable (GJ-transferable) dye calcein deep red acetate (see Supplemental Methods; supplemental material available online with this article; https://doi.org/10.1172/jci.insight.162178DS1), while recipient ERVMs expressing GFP or GFP-Kin1-CA were labeled with the GJ-nontransferable dye CytoTell Red 590 (see Supplemental Methods). ERVMs expressing GFP-Kin1-CA displayed a significantly increased rate of calcein deep red acetate dye transfer compared with ERVMs expressing control GFP, following mixing of the respective donor and recipient cell populations (Figure 2B), suggesting that overexpression of GFP-Kin1-CA enhances GJ-mediated cell communication. Taken together, these results implicate obscurin-kin1 in the modulation of cardiomyocyte mechanochemical coupling, likely by directly regulating the phosphorylation status of N-cadherin and therefore its adhesive and mechanical properties.

### Obscurin-kin1 phosphorylates N-cadherin at Ser-788.

To test our hypothesis that obscurin-kin1 modulates the mechanochemical coupling of cardiomyocytes via phosphorylation of N-cadherin, we set forth to identify the potential phosphorylation target site(s) of obscurin-kin1 within the cytoplasmic domain of N-cadherin. To this end, we used the baculovirus system to produce kin1-CA coupled to a 6×His tag (His-Kin1-CA; Supplemental Figure 1A) and the bacterial system to produce 2 different N-cadherin constructs tagged to 6×His containing either the entire cytoplasmic domain (aa 746–906) (accession no. P15116, https://www.uniprot.org/uniprot; His-Ncad_746–906_, Supplemental Figure 1A), which we previously identified as the binding site of obscurin-kin1, or a smaller portion within the cytoplasmic domain (aa 786–880, accession no. P15116; His-Ncad_786–880_, Supplemental Figure 1A) that is enriched in Ser residues, which are predicted to be potential phosphorylation target sites by EXPASY (https://www.expasy.org/)/GPS2.1 (Group-based Prediction System version 2.1: enhanced prediction of kinase-specific phosphorylation sites; https://gps.biocuckoo.cn/). Following affinity purification, His-Ncad_746–906_ and His-Ncad_786–880_ were subjected to in vitro kinase assays in the presence of His-Kin1-CA or control baculovirus preparation infected with empty vector that had undergone the same purification process as His-Kin1-CA. The reaction mixtures were subsequently subjected to liquid chromatography–tandem mass spectrometry (LC-MS/MS) analysis. An N-cadherin peptide encompassing amino acids 786–808 containing a single phosphorylation event at Ser-788 was repeatedly identified in both the His-Ncad_746–906_ (Supplemental Figure 1B) and the His-Ncad_786–880_ (Supplemental Figure 1C) reaction mixtures when treated with His-Kin1-CA, but not with the control preparation. Sequence alignment using Clustal Omega software (https://www.ebi.ac.uk/Tools/msa/clustalo/) indicated that Ser-788 is highly conserved among N-cadherin orthologs across various species as well as among different members of the cadherin superfamily (Supplemental Figure 1D), suggesting that the identified phosphorylation event is of potential (patho)physiological relevance across different organisms and possibly within the cadherin superfamily.

**Figure 3:**
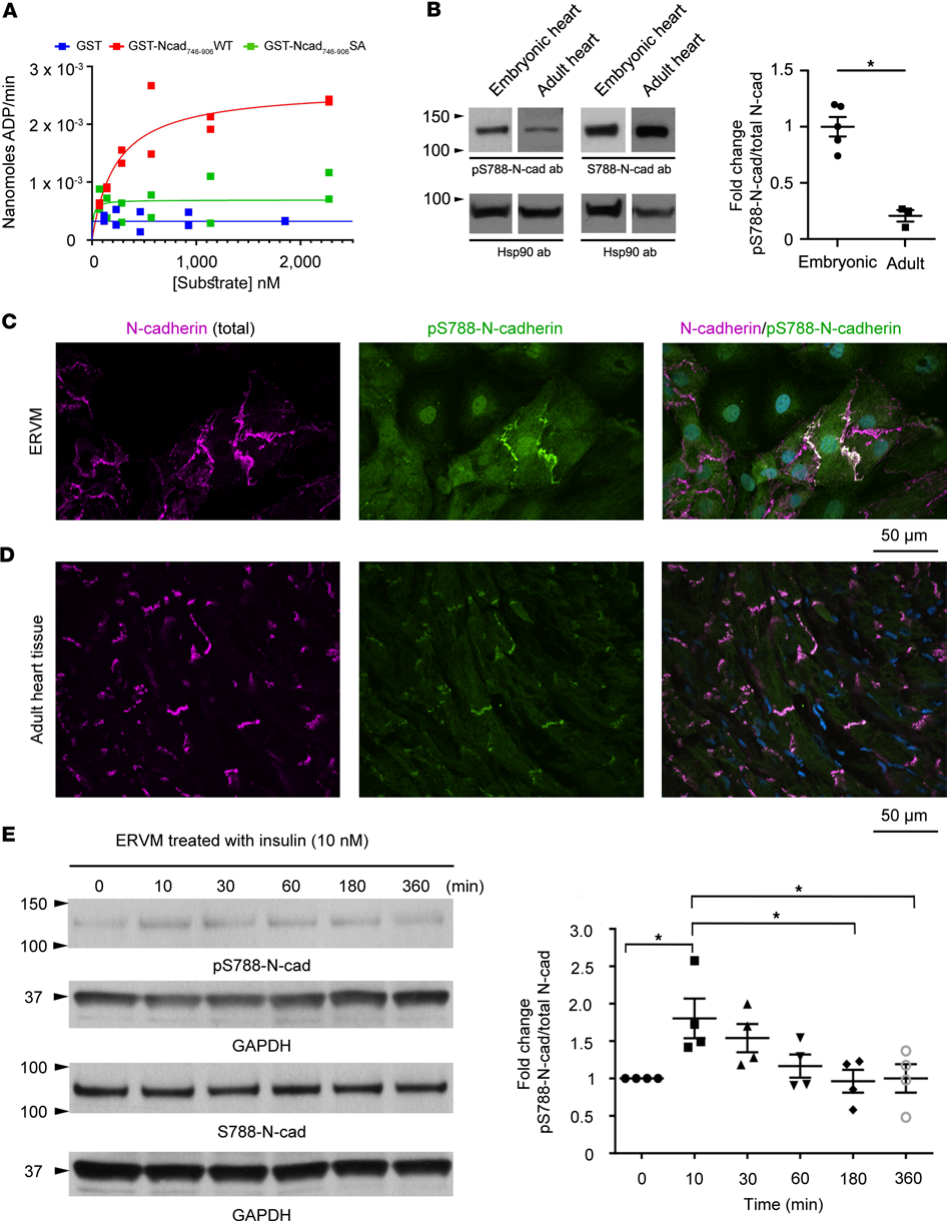
Obscurin-kin1 phosphorylates N-cadherin at Ser-788. (**A**) Michaelis-Menten plot showing the nanomoles of ADP produced per minute at constant amount of His-Kin1-CA (26 nM) and ATP (100 μM) and varying substrate concentrations (70–2272 nM). An apparent *V_max_* (2.63 × 10^–3^ nanomoles ADP/min), *K_M_* (232.8 nM), and *K_cat_* (5.05 min^–1^) for GST-Ncad_746–906_-WT were calculated, while use of GST and GST-Ncad_746–906_-SA did not yield any appreciable ADP formation. Values from 2 technical repeats at each substrate concentration are shown. (**B**) Representative immunoblots using custom-made affinity-purified phospho-Ser-788 N-cadherin (pS788-N-cad) and total Ser-788 (S788-N-cad) antibodies with protein lysates prepared from E21 and adult (3-month-old) rat hearts. Although baseline phosphorylation of N-cadherin at Ser-788 was observed in both embryonic and adult hearts, the former contain a 2-fold higher ratio of pS788-N-cad/total N-cad compared with the latter. Quantification was performed following normalization to Hsp90 that was used as loading control. Noncontinuous lanes are separated with white space (please see supplemental material); *n* = 5 biological samples for embryonic rat hearts and *n* = 3 biological samples for adult rat hearts run in duplicate. Unpaired, 2-tailed Mann-Whitney *t* test, *P = 0.0357. (**C** and **D**) Immunofluorescent staining of ERVM cultures (**C**) and 1-month-old mouse myocardia (**D**) with pS788-N-cad and total N-cadherin antibodies followed by confocal evaluation showed the presence of S788-N-cad at cell junctions and ICDs, respectively; ERVM cultures and tissue sections were counterstained with Hoechst to identify nuclei. Scale bars: 50 μm. (**E**) Immunoblotting of ERVM protein lysates with pS788-N-cad and S788-N-cad antibodies following treatment with 10 nM insulin indicated a statistically significant increase in the phosphorylation levels of N-cadherin at Ser-788 at 10 minutes, which returned to pretreatment levels by 180 minutes; quantification of the pS788-N-cad/total N-cad ratio at the indicated time points was performed following normalization to GAPDH that was used as loading control with the pS788-N-cad/total N-cad ratio at 0 minutes set to 1 and ratios at remaining time points expressed relative to it; *n* = 4. One-way ANOVA followed by Tukey’s multiple-comparison test, *P = 0.0486 (10 minutes vs. 0 minutes), *P = 0.0365 (10 minutes vs. 180 minutes), and *P = 0.0491 (10 minutes vs. 360 minutes). Data are expressed as mean ± SEM.

To investigate the kinetics of the His-Kin1-CA/N-cadherin–Ser-788 phosphorylation reaction, we used a fluorometric assay to assess the apparent *K_M_* and *V_max_*. We evaluated the His-Kin1-CA enzyme kinetics by varying the concentration of recombinant GST-Ncad_746–906_ (substrate-1) between 70 and 2272 nM and maintaining the amount of ATP (substrate-2) constant at 100 μM. Our analysis yielded an apparent *K_M_* of 232.8 nM and a *V_max_* of 2.63 × 10^–3^ nmol ADP/min resulting in a *K_cat_* of 5.05 min^–1^, indicative of an efficient phosphorylation reaction (Figure 3A); of note, similar catalytic turnover rates were independently obtained in parallel experiments (Supplemental Figure 2). Importantly, use of control GST protein or phosphoablated GST-Ncad_746–906_ in which Ser-788 was replaced by Ala showed lack of phosphorylation of either recombinant protein by His-Kin1-CA (Figure 3A and Supplemental Figure 2), further supporting the specificity of our findings.

To confirm the presence of phosphorylation on Ser-788 in situ, we generated phospho-specific antibodies, which we tested in immunoblots of protein lysates prepared from rat embryonic day 21 (E21) and adult hearts. We detected baseline phosphorylation of Ser-788 in both embryonic and adult cardiac lysates (Figure 3B), albeit to different extents, with the latter exhibiting approximately 2-fold lower levels, suggesting that Ser-788 phosphorylation may play a more potent role during embryonic heart development. Using immunofluorescence combined with confocal optics, we further confirmed the presence of phospho-Ser-788 N-cadherin at cell junctions in developing ERVMs (Figure 3C) and the ICD in adult myocardium (Figure 3D).

Given the earlier observations by Lee et al. indicating that N-cadherin undergoes further phosphorylation upon stimulation (32), we next investigated whether phosphorylation of Ser-788 responds to growth stimulation. We therefore treated ERVM cultures with 10 nM insulin and examined the effects of this treatment at regular intervals over a period of 360 minutes. We observed a statistically significant increase (~2-fold) in the levels of Ser-788 phosphorylation 10 minutes after treatment compared with control (0 minutes), which gradually returned to basal levels (Figure 3E), indicating that the obscurin-kin1/N-cadherin-Ser-788 phosphorylation axis may be modulated by responses elicited through activation of the insulin receptor, which plays a key role in the regulation of cardiac growth, survival, and metabolism (35).

### N-cadherin phosphorylation on Ser-788 modulates cardiomyocyte adhesion and communication.

**Figure 4:**
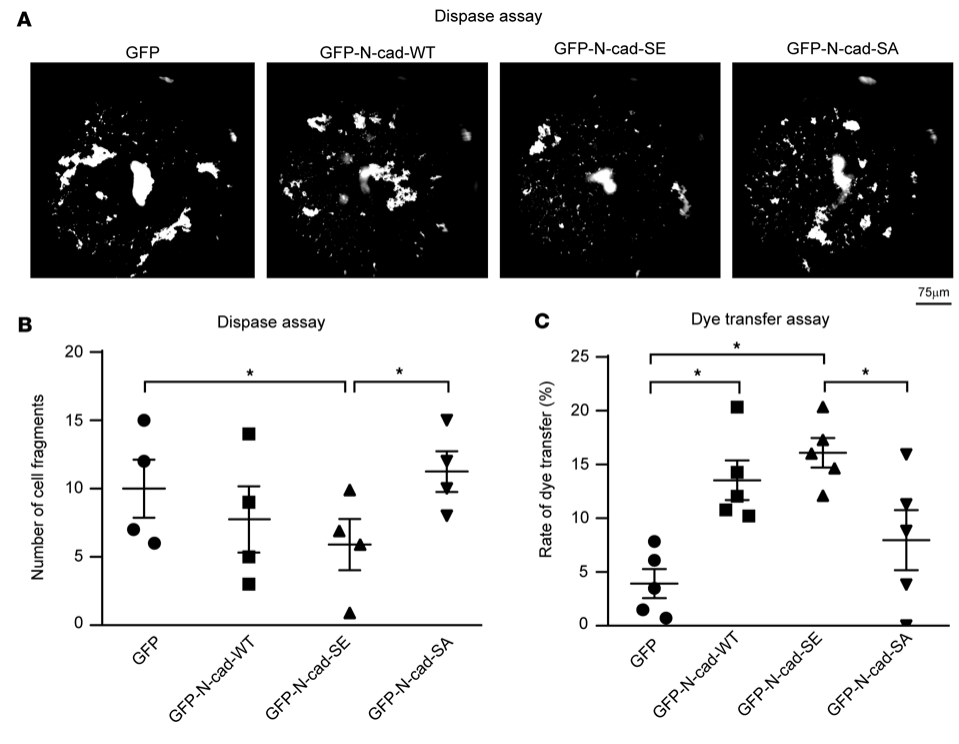
Phosphorylation of N-cadherin at Ser-788 plays a key role in cardiomyocyte adhesion and communication. (**A** and **B**) Overexpression of phosphomimic GFP-N-cad-SE, but not control GFP protein or phosphoablated GFP-N-cad-SA, in ERVMs significantly increased cardiomyocyte adhesion as indicated by the reduced number of cell fragments (≥100 mm) obtained in a Dispase assay; of note, GFP-N-cad-WT shows a clear trend toward increased mechanical coupling; *n* = 4. Scale bar: 75 μm. Repeated measures 1-way ANOVA followed by Fisher’s least significant difference (LSD) post hoc test, *P = 0.028 for comparison of GFP-N-cad-SE vs. GFP and *P = 0.0036 for comparison of GFP-N-cad-SE vs. GFP-N-cad-SA. (**C**) Overexpression of phosphomimic GFP-N-cad-SE, but not control GFP protein or phosphoablated GFP-N-cad-SA, in ERVMs significantly enhances intercellular communication as shown by the increased rate of dye transfer; interestingly, overexpression of GFP-N-cad-WT elicits the same effect, suggesting that it may be phosphorylated by endogenous kin1 present in obscurin-B (*n* = 5). Repeated measures 1-way ANOVA followed by Tukey’s multiple-comparison test, *P = 0.008 for comparison of GFP-N-cad-SE vs. GFP, *P = 0.0386 for comparison of GFP-N-cad-SE vs. GFP-N-cad-SA, and *P = 0.0023 for comparison of GFP-N-cad-WT vs. GFP. Data are expressed as mean ± SEM.

We next investigated whether the effects of obscurin kin1-CA overexpression on the mechanochemical properties of cardiomyocytes are mediated through N-cadherin phosphorylation at Ser-788. To this end, we generated GFP-tagged wild-type, phosphomimic, and phosphoablated full-length N-cadherin constructs, referred to as GFP-N-cad-WT and GFP-N-cad-SE in which Ser-788 was substituted with glutamic acid (Glu, E), and GFP-N-cad-SA in which Ser-788 was substituted with alanine (Ala, A), respectively. Following transfection of these constructs in ERVMs, GFP-positive cells were collected via cell sorting and subsequently subjected to Dispase mechanical dissociation (Figure 4, A and B) and dye transfer assay (Figure 4C). Overexpression of GFP-N-cad-SE resulted in significantly decreased cell fragmentation (Figure 4, A and B) and an increased rate of dye transfer following mixing of the respective donor and recipient cell populations (Figure 4C), compared with control GFP and GFP-N-cad-SA, indicating that phosphorylation of N-cadherin at Ser-788 enhances cardiomyocyte adhesion and communication. Notably, similarly to GFP-N-cad-SE, overexpression of GFP-N-cad-WT resulted in a clear trend toward reduced cell fragmentation (Figure 4, A and B) and a significantly increased rate of dye transfer (Figure 4C), compared with control GFP and GFP-N-cad-SA, suggesting that exogenous GFP-N-cad-WT is likely phosphorylated by endogenous kin1 present in obscurin-B in ERVMs.

Together, these findings demonstrate that overexpression of obscurin-Kin1-CA (Figure 2) and phosphomimic N-cadherin (Figure 4) elicit the same effects on cardiomyocyte adhesion and communication. This strongly suggests that obscurin-kin1 modulates the mechanochemical properties of cardiomyocytes through phosphorylation of N-cadherin on Ser-788 located in the juxtamembrane region of its cytoplasmic domain.

### N-cadherin phosphorylation at Ser-788 modulates binding to p120-catenin.

**Figure 5:**
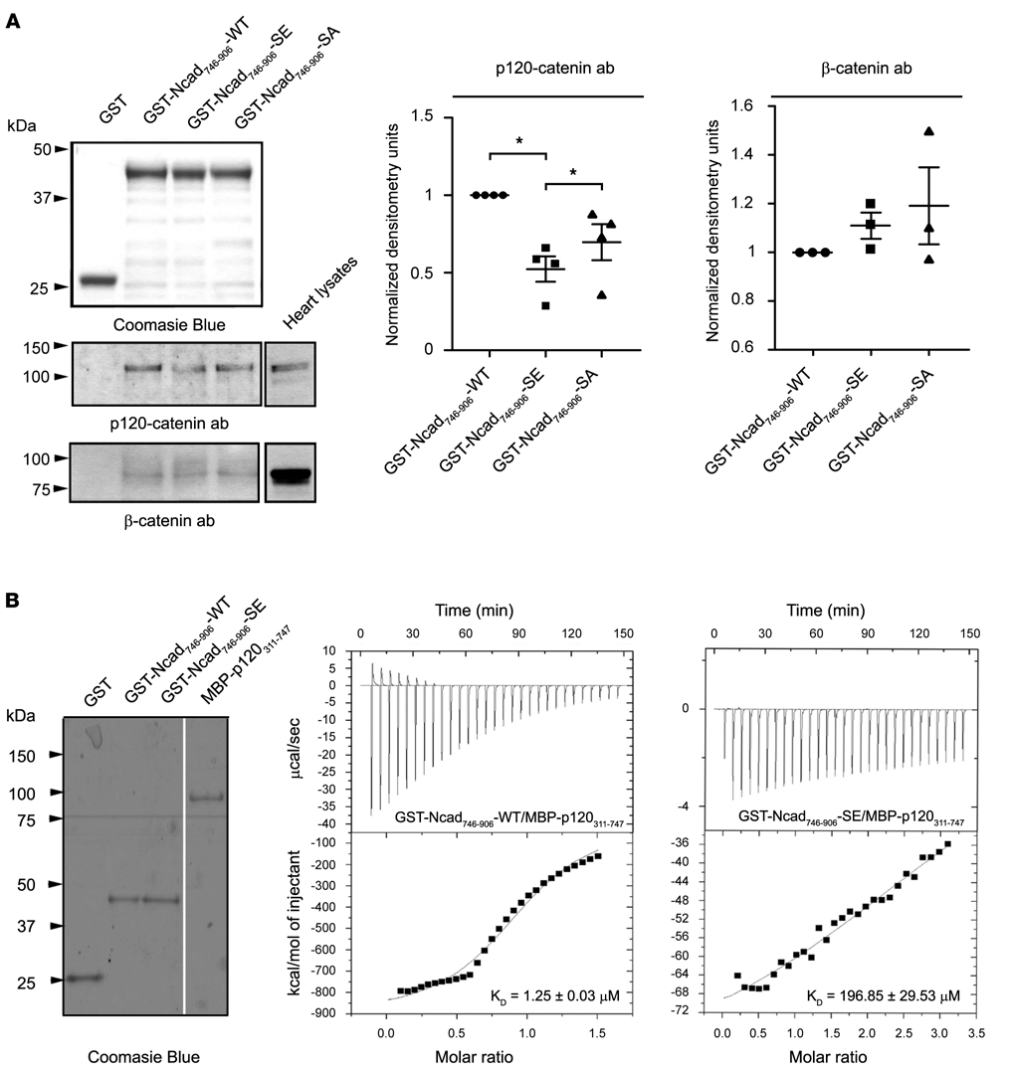
Phosphorylation of N-cadherin at Ser-788 results in reduced binding to P120-catenin. (**A**) Equivalent amounts of recombinant GST-tagged WT (GST-Ncad_746–906_-WT), phosphomimic (GST-Ncad_746–906_-SE), and phosphoablated (GST-Ncad_746–906_-SA) N-cadherin proteins containing the cytoplasmic domain along with control GST protein, as shown by Commassie Blue staining, were tested in pull-down assays for their ability to precipitate and retain endogenous p120-catenin from adult mouse cardiac lysates. Phosphomimic GST-Ncad_746–906_-SE exhibited significantly reduced binding to p120-catenin compared with GST-Ncad_746–906_-WT and phosphoablated GST-Ncad_746–906_-SA proteins (*n* = 4); the binding of p120-catenin to GST-Ncad_746–906_-WT was set to 1, while binding of p120-catenin to either GST-Ncad_746–906_-SE or GST-Ncad_746–906_-SA was normalized to GST-Ncad_746–906_-WT. Statistical evaluation was performed with repeated measures 1-way ANOVA followed by Tukey’s multiple-comparison test, *P = 0.0203 for comparison of GST-Ncad_746–906_-SE vs. Ncad_746–906_-WT and *P = 0.0496 for comparison of GST-Ncad_746–906_-SE vs. GST-Ncad_746–906_-SA. This effect was specific to p120-catenin, as binding of β-catenin to GST-Ncad_746–906_-WT, GST-Ncad_746–906_-SE, or GST-Ncad_746–906_-SA was unaltered (*n* = 3). Statistical significance was determined with repeated measures 1-way ANOVA followed by Tukey’s multiple-comparison test. Noncontinuous lanes are separated with white space (please see supplemental material). (**B**) Coomassie Blue staining showing recombinant GST, GST-Ncad_746–906_-WT, GST-N-cad_746–906_-SE, and MBP-p120-catenin_311–747_ that were used in ITC experiments; noncontinuous lanes are separated with white space (please see supplemental material). An average binding affinity, *K_D_*, of 1.25 ± 0.03 μM was determined for GST-Ncad_746–906_-WT and MBP-p120-catenin_311–747_, whereas a *K_D_* of 196.85 ± 29.53 μM was calculated for GST-Ncad_746–906_-SE and MBP-p120-catenin_311–747_, consistent with the decreased binding observed in the pull-down assays; *n* = 3 independent ITC experiments. Data are expressed as mean ± SEM.

Intracellularly, N-cadherin links the underlying actin cytoskeleton to the cell membrane via its direct and indirect interactions with a group of adaptor proteins, called catenins (25). P120-catenin binds to the juxtamembrane region (aa 774–790) of N-cadherin containing Ser-788 (36). We therefore examined whether phosphorylation of N-cadherin at Ser-788 regulates its direct binding using GST pull-down assays. To this end, equivalent amounts of GST-tagged WT (GST-Ncad_746–906_-WT), phosphomimic (GST-Ncad_746–906_-SE), and phosphoablated (GST-Ncad_746–906_-SA) recombinant proteins containing the cytoplasmic domain of N-cadherin along with control GST were immobilized on a glutathione sepharose column via their GST tag and incubated with protein lysates prepared from adult mouse left ventricles (Figure 5A). GST-Ncad_746–906_-SE exhibited significantly reduced binding to endogenous p120-catenin compared with GST-Ncad_746–906_-WT and GST-Ncad_746–906_-SA (Figure 5A), implicating Ser-788 phosphorylation in the regulation of the N-cadherin/p120-catenin binding. The specificity of this finding was corroborated by the lack of an effect on the binding of β-catenin to GST-Ncad_746–906_-SE (Figure 5A), which binds to the extreme COOH terminus of N-cadherin (aa 856–885) (37).

To quantitatively evaluate the effect of Ser-788 phosphorylation on the N-cad/p120-catenin binding, we performed isothermal calorimetry (ITC) assays. GST-Ncad_746–906_-WT and GST-Ncad_746–906_-SE served as ligands, while the middle portion of p120-catenin containing armadillo domains 1–8 (p120-catenin_311–747_) that have been previously implicated in cadherin binding (38), fused to maltose binding protein (MBP) (Figure 5B) served as sample macromolecule. An average binding affinity, *K_D_*, of 1.25 ± 0.03 μM was determined for the GST-Ncad_746–906_-WT and p120-catenin_311–747_ proteins, indicative of a moderate and dynamic interaction (Figure 5B). Remarkably, binding between GST-Ncad_746–906_-SE and p120-catenin_311–747_ was dramatically weaker, with an average *K_D_* of 196.85 ± 29.53 μM (Figure 5B), corroborating the significantly reduced binding seen in the GST pull-down assay (Figure 5A).

### Molecular dynamics simulation of phosphorylation of N-cadherin at Ser-788 and impact on RhoA activity.

Earlier work has demonstrated that p120-catenin binding to cadherins modulates their targeting to and stability in cell junctions, and therefore their adhesive properties (36). In particular, p120-catenin binding has been implicated in preventing clathrin-mediated endocytosis of cadherins and thus degradation via the lysosomal pathway (39). This process has been suggested to be mediated via a highly conserved acidic cluster of amino acid residues (i.e., DEE, aa 774–776 in N-cadherin) present in the core of the p120-catenin binding site on cadherins that functions as an endocytic signal when exposed, due to reduced binding of p120-catenin (40).

**Figure 6:**
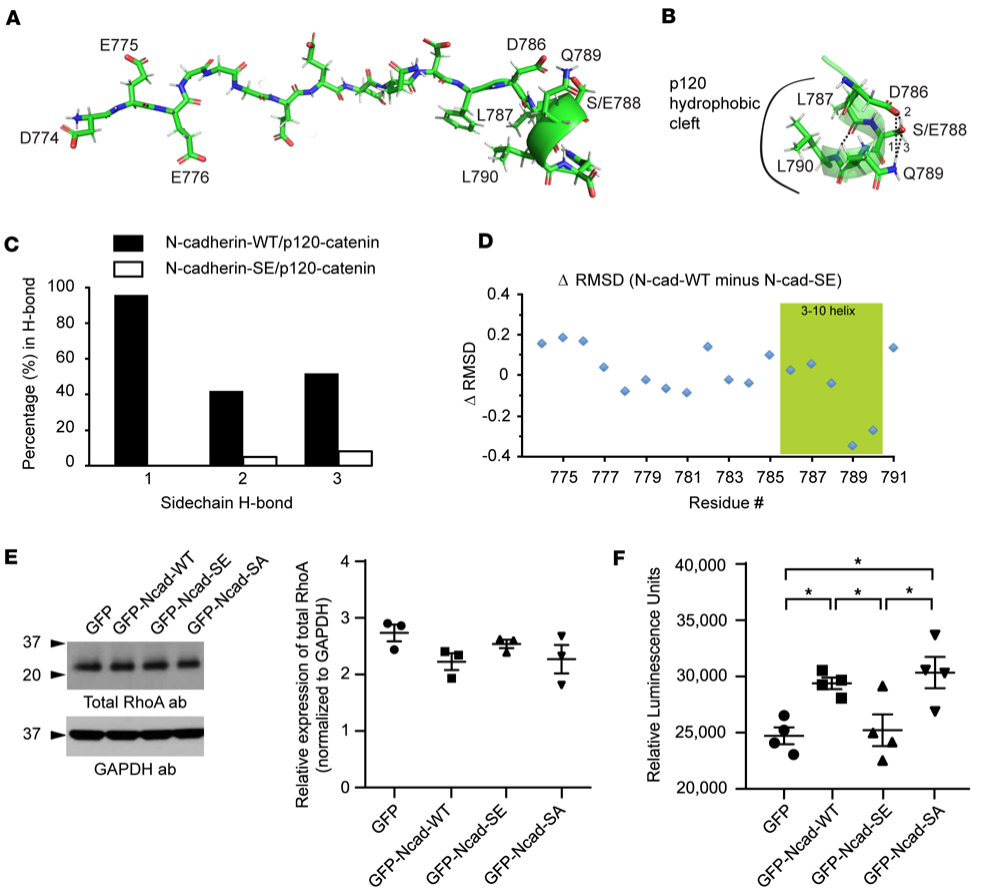
Impact of the N-cadherin/p120-catenin binding on RhoA activity. (**A**) Model of the p120-catenin–bound N-cadherin D774–H791 fragment, based on the p120-catenin/E-cadherin structure (PDB: 3L6X), showing an extended conformation with a COOH-terminal 3-10 helix. (**B** and **C**) When the phosphomimic E778 is included in molecular dynamic simulations of this p120-catenin/N-cadherin complex, the 3-10 helix that supports hydrophobic interactions with p120-catenin loses multiple stabilizing hydrogen bonds. (**D**) This loss of hydrogen bonds destabilizes the helix, resulting in more motion and weaker interactions with the corresponding hydrophobic patch on p120-catenin, relative to simulations performed on the WT complex. (**E**) Representative immunoblot using protein lysates prepared from HEK293 cells transfected with control GFP or full-length WT (GFP-Ncad-WT), phosphomimic (GFP-Ncad-SE) or phosphoablated (GFP-Ncad-SA) N-cadherin constructs indicate similar levels of total RhoA across all groups; GAPDH was used as loading control (*n* = 3). Data are expressed as mean ± SEM and 1-way ANOVA followed by Fisher’s LSD test was used for statistical evaluation. (**F**) Measurement of GTP-RhoA levels (i.e., active RhoA) using luminescence-based G-LISA indicated that overexpression of phosphoablated GFP-Ncad-SA in HEK293 cells results in significantly increased RhoA activity compared with GFP and GFP-Ncad-SE; of note, GFP-Ncad-WT behaves similarly to GFP-Ncad-SA; *n* = 4 experiments performed in duplicate. Data are expressed as mean ± SEM and statistical evaluation was done with 1-way ANOVA followed by a Fisher’s LSD test, *P = 0.0104 for GFP vs. GFP-Ncad-WT, *P = 0.0189 for GFP-Ncad-WT vs. GFP-Ncad-SE, *P = 0.006 for GFP-Ncad-SE vs. GFP-Ncad-SA, and *P = 0.0033 for GFP vs. GFP-Ncad-SA.

To explore how phosphorylation of N-cadherin at Ser-788 and ultimately the addition of a negative charge by the phosphate group affects the conformation of the N-cadherin/p120-catenin complex and thus the exposure of the DEE motif, we performed molecular dynamics simulations using phosphomimic-SE (Figure 6) or phospho-Ser-788 (Supplemental Figure 3) N-cadherin based on a model of the existing high-resolution human E-cadherin/p120-catenin complex (PDB: 3L6X) (Figure 6A). Of note, the relevant E- and N-cadherin peptides (carrying the p120-catenin binding site) are similar in sequence, with the only changes involving residues Phe-785 and His-791, which are replaced by Tyr and Gln in N-cadherin, respectively. Given that both substitutions are solvent facing in the N-cadherin/p120 complex structure, they do not significantly alter any binding interactions upon model equilibration. Analysis of our models indicated that the NH_2_-terminal region of the N-cadherin peptide binds p120-catenin through a series of electrostatic interactions, as described in Ishiyama et al. (41). These remained unperturbed between the WT and phosphomimic-SE or phospho-Ser-788 N-cadherin/p120-catenin simulations. Conversely, the COOH terminus of the N-cadherin peptides binds to p120-catenin primarily through hydrophobic interactions, made possible by N-cadherin residues 786–790 adopting a short 3-10 helix (Figure 6B). This helix is stabilized internally by several side-chain hydrogen bonds, including residues Asp-786 and Ser-788, Asp-786 and Gln-789, and Ser-788 and Gln-789. In the WT simulation, these interactions were present 96%, 42%, and 52% of the time, respectively. In contrast, in the phosphomimic-SE (Figure 6C) and phospho-Ser-788 (Supplemental Figure 3A) simulations, these bonds were present 0%, 15%, and 8% and 4%, 0%, and 14% of the time, respectively. This loss of stabilizing interactions results in increased mobility in this 3-10 helix; this is evidenced by the SE-phosphomimic and phospho-Ser-788 N-cadherin variants having a larger root mean squared deviation (ΔRMSD = 0.31 Å for phosphomimic-SE and ΔRMSD = 0.13 Å for phospho-Ser-788) in this area compared with WT, with no concomitant ΔRMSD change in the rest of the peptide (Figure 6D and Supplemental Figure 3B). This suggests that adding a negative charge to residue Ser-788 (via phosphorylation) destabilizes the binding-competent conformation of N-cadherin to p120-catenin, consistent with the reduction in p120-catenin affinity determined by the pull-down assays and ITC (Figure 5). Interestingly though, the addition of a negative charge on Ser-788 fails to expose the DEE endocytic signature motif, implying that phosphorylation of N-cadherin at Ser-788 by obscurin-kin1 does not modulate its stability at the plasma membrane. In agreement with these findings, overexpression of GFP-tagged full-length WT (GFP-Ncad-WT), SE phosphomimic (GFP-Ncad-SE), or SA phosphoablated (GFP-Ncad-SA) N-cadherin proteins in HEK293 cells indicated that they were expressed at similar levels (Supplemental Figure 4A) and preferentially concentrated at the plasma membrane, in contrast with control GFP-protein that accumulated in the nucleus and perinuclear region, likely due to its small size (Supplemental Figure 4, B–E).

Previous work has indicated that in addition to regulating cadherin stability at the plasma membrane, p120-catenin modulates the activity of small GTPases; in particular, it inhibits RhoA activity, which has been implicated in cadherin clustering, an early event in the assembly of cell junctions, and actin remodeling (39). Accordingly, it has been proposed that the ability of p120-catenin to modulate Rho GTPase activity may be inhibited by cadherin binding (39). We therefore examined whether reduced binding of phosphorylated N-cadherin at Ser-788 to p120-catenin may affect RhoA activity. To this end, we used HEK293 cells to overexpress GFP-tagged full-length WT, phosphomimic-SE, and phosphoablated-SA N-cadherin proteins along with control GFP and performed G-LISA assays to evaluate RhoA activity levels. Interestingly, while the expression levels of total RhoA were unaltered across control and experimental groups (Figure 6E), we found that RhoA activity was significantly increased following overexpression of phosphoablated-SA N-cadherin compared with control GFP protein and phosphomimic-SE N-cadherin (Figure 6F), implicating Ser-788 phosphorylation in the regulation of RhoA-mediated processes via the dynamic binding of p120-catenin to N-cadherin and RhoA. Notably, overexpression of phosphomimic-SE N-cadherin did not induce a further decline in RhoA activity relative to control GFP, in agreement with the inherently low levels of endogenous RhoA (https://www.proteinatlas.org/), and therefore activity, in HEK293 cells. Moreover, WT N-cadherin behaved similarly to phosphoablated-SA N-cadherin, exhibiting increased RhoA activity compared with control GFP and phosphomimic-SE N-cadherin, consistent with the relatively low levels of endogenous obscurin in HEK293 cells (https://www.proteinatlas.org/), implying lack of substantial phosphorylation of exogenous WT N-cadherin by endogenous obscurin-kin1.

### Upregulation of the obscurin-B/N-cadherin phosphorylation axis in heart failure.

**Figure 7:**
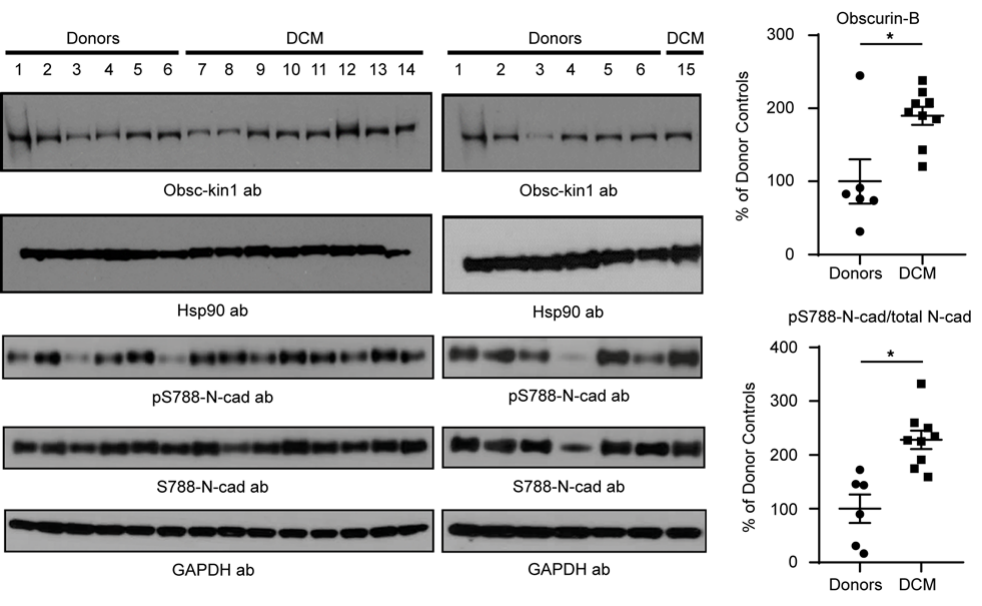
The obscurin-kin1/phospho-N-cadherin axis is upregulated in end-stage heart failure. Immunoblot analysis of protein lysates prepared from donor (lanes 1–6) and end-stage heart failure DCM (lanes 7–15) biopsies using antibodies against obscurin-kin1, phospho-N-cadherin-S788, and total N-cadherin-S788; GAPDH was used as loading control. Densitometric evaluation of the respective immunoreactive bands indicated that the levels of obscurin B containing kin1 and the ratio of phospho-N-cadherin-S788 to total N-cadherin were significantly upregulated in DCM samples compared with donor controls, following normalization to GAPDH that served as loading control; *n* = 6 control donor biopsies and *n* = 9 DCM biopsies run in triplicate. Data are expressed as mean ± SEM. Unpaired, 2-tailed *t* test followed by Mann-Whitney post hoc test, *P = 0.036 for obscurin-B and *P = 0.0008 for phospho-N-cadherin-S788/total N-cadherin ratio.

Given the documented involvement of the *OBSCN* gene in the development of cardiomyopathies (19), we next interrogated whether the expression levels of obscurin-B containing kin1 and phospho-Ser-788 N-cadherin are altered in human end-stage heart failure DCM samples (Table 1). Of note, the expression profile of obscurin-B in cardiac muscle has been controversial, with early reports suggesting that it is primarily expressed in skeletal muscle and to a lesser extent in cardiac muscle, which was postulated to mainly contain small obscurin-kinase isoforms (22). To address this issue and given that most studies have used rodent myocardia, we performed reverse transcription PCR (RT-PCR) analysis using human donor left ventricle (LV) samples as well as mouse myocardia to specifically amplify giant obscurin-B. Our findings indicated that in contrast with mouse myocardia that contain minute amounts of obscurin-B transcripts (Supplemental Figure 5) and protein under normal conditions (4, 11, 17), human donor LVs express adequate amounts of obscurin-B transcripts (Supplemental Figure 5), consistent with earlier studies reporting that obscurin-B appears to be the predominant isoform in human heart (42, 43). We therefore set forth to examine whether the levels of obscurin-B containing kin1 and phospho-Ser-788 N-cadherin were altered in human DCM end-stage heart failure LV samples compared with donor controls using antibodies against obscurin-kin1, phospho-Ser-788, and total N-cadherin (Figure 7). Importantly, we observed a significant increase in both the levels of obscurin-B containing kin1 and phospho-Ser-788 N-cadherin in DCM samples relative to controls, indicating that the obscurin-kin1/phospho-Ser-788 N-cadherin axis is upregulated in human end-stage heart failure.

## Discussion

Obscurins comprise a family of giant cytoskeletal regulators that are abundantly expressed in striated muscle cells, localizing in diverse subcellular compartments (2, 3). Obscurin-kinase isoforms preferentially accumulate at the ICD in adult cardiac muscle (24), the unique microdomain of the sarcolemma that contributes to the mechanochemical coupling of adjacent cardiomyocytes (26). Consistent with this, earlier work from our group indicated that obscurin-kin1 specifically binds to and phosphorylates the cytoplasmic domain of N-cadherin in vitro (24). N-cadherin is a major component of the AJ, mediating not only mechanical adhesion, but also contributing to the electrical coupling of adjoining cardiomyocytes and partaking in the organization of the cortical cytoskeleton through its interactions with a group of accessory proteins, collectively known as catenins (29, 44). Consistent with this, cardiac-specific loss of N-cadherin during adulthood leads to disassembly of the ICD, myofibrillar disarray, and DCM accompanied by ventricular arrhythmia and eventually sudden cardiac death (29).

Our current studies demonstrate that obscurin-B containing kin1 and N-cadherin codistribute at contact sites in developing cardiomyocytes and their codistribution is modulated by Ca^2+^, highlighting the physiological relevance of the obscurin-kin1 regulation of N-cadherin. Accordingly, our MS studies identified Ser-788, located in the juxtamembrane region of the cytoplasmic domain of N-cadherin, as the target site of obscurin-kin1. Of note, smaller obscurin-kinase isoforms containing either both kin1 and kin2 (double kinase) or only kin2 (single kinase) have been described (22, 45); however, their exact molecular identity and spatiotemporal distribution have remained elusive. Interestingly, Hsu et al. reported phosphorylation of N-cadherin at Ser-788 in mouse embryonic fibroblasts in response to insulin treatment in a comprehensive phosphoproteomics analysis, yet without identifying the responsible kinase or the physiological significance of this modification (46).

Twelve Ser/Thr kinases have been reported to modulate phosphorylation of AJ proteins, with all of them residing in the cytoplasm, that are likely recruited to AJ upon activation (47). In contrast with the identified cytoplasmic Ser/Thr kinases, which are suggested to transiently localize to AJ, a population of obscurin-B molecules containing kin1 appear to be permanently found at the ICD of cardiomyocytes. Although the activation mechanism of obscurin-kin1 is currently unknown, our studies showed that treatment of embryonic cardiomyocytes with insulin results in a rapid, transient upregulation of Ser-788 phosphorylation. Interestingly, bioinformatics analysis (https://www.phosphosite.org/homeAction.action) predicted the presence of phosphorylation within the catalytic portion of kin1, which is in agreement with its ability to undergo autophosphorylation in vitro (24). Moreover, Fleming et al. reported that the linker region between kin1 and kin2 may contain several phosphorylation sites, with at least some of them being (direct) targets of kin1, suggesting that such events may modulate kin1 (and possibly kin2) activity (48). In support of these observations, obscurin was recently identified as a kinase-bearing cytoskeletal protein that undergoes extensive phosphorylation in response to exercise and maximum-intensity contractions in striated muscles, implicating it in hypertrophic and mechanotransduction responses (49, 50). Consistent with the notion that (auto)phosphorylation may play a key role in kin1’s regulation, the NH_2_-terminal Ser/Thr kinase domain (SK1) of striated muscle preferentially expressed gene (*SPEG*), which arose from *OBSCN* via gene duplication (23), is capable of autophosphorylation, while PKB/AKT directly phosphorylates the interkinase region of SPEG in response to insulin treatment, resulting in activation of the COOH-terminal SPEG kinase, SK2 (51). Although it is currently unknown whether the enzymatic activity of obscurin-kin1 is also regulated by PKB/AKT, our earlier studies have shown that obscurin is intimately associated with the PI3K-PKB/AKT-mTOR pathway in both cardiomyocytes (4) and epithelial cells (13), and alteration of their association has major effects in key cellular processes, including growth, adhesion, and motility/contractility.

Ectopic expression of catalytically active obscurin-kin1 in ERVMs resulted in enhanced cell adhesion and communication. Although it is likely that obscurin-kin1 has additional targets in cardiomyocytes (24), it is reasonable to assume that the effects of kin1 in the modulation of the mechanochemical coupling of cardiomyocytes are mediated (at least in part) via phosphorylation of N-cadherin at Ser-788. In accordance with this, overexpression of phosphomimic N-cadherin in embryonic cardiomyocytes elicited similar effects. Notably, baseline phosphorylation of Ser-788 was considerably higher in embryonic compared with adult cardiomyocytes, suggesting that it may have a more prominent role during cardiac development and differentiation when cell junction assembly takes place (39), rather than in adulthood where intercellular connections have already formed.

The molecular mechanisms that regulate the functional properties of N-cadherin have only been cursorily examined. Accordingly, Lee et al. demonstrated that N-cadherin is a target of endogenous kinases and phosphatases, and that its phosphorylation state is an important determinant of its stability and functionality at the cell surface (32). Moreover, the same authors reported that native N-cadherin contains high basal levels of Ser phosphorylation in the heart, retina, brain, and lens, whereas upon stimulation it undergoes further Ser and Tyr phosphorylation (32). Consistent with this, Qi et al. documented that phosphorylation of N-cadherin by Src-kinase on Tyr-860 abolishes its ability to bind β-catenin at the plasma membrane of melanoma cells, inhibiting their transmigration across the endothelium (33), while Tyr phosphorylation of N-cadherin promotes its proteolytic cleavage and diminishes cell adhesion (32). While the extracellular domain of N-cadherin mediates cell adhesion in a homophilic way in a Ca^2+^-dependent manner, its cytoplasmic domain is also involved in the construction of tight and compact cell junctions by supporting interactions with the underlying actin cytoskeleton (52, 53). Such binding interactions involve the formation of a dynamic multiprotein complex consisting of linker proteins (e.g., catenins), GTPases (e.g., RhoA), ubiquitin ligases (e.g., Hakai ligase), kinases (e.g., PIPKIγ and obscurin-kin1 as our data indicate), and phosphatases (e.g., PTPσ) (54, 55).

Previous work has demonstrated that p120-catenin binds to the juxtamembrane region (aa 774–790) of N-cadherin (39) that contains Ser-788. This region of N-cadherin, which is normally disordered, is anchored to p120-catenin via electrostatic interactions involving the Asp-774–Glu-775–Glu-776 (DEE) motif and hydrophobic interactions encompassing residues Leu-787, Ser-788, Gln-789, and Leu-790 (41). Asp-774 and Leu-790 are separated by more than 3 nm in the N-cadherin–bound p120-catenin form, and there is no obvious mechanism through which phosphorylation of Ser-788 would directly influence the structure, dynamics, and binding capacity of the DEE motif. Instead, Ser-788 phosphorylation disrupts the local N-cadherin secondary structure. This in turn appears to destabilize the hydrophobic interactions between the juxtamembrane region of N-cadherin and p120-catenin, likely leading to peeling away of N-cadherin from the p120-catenin binding groove, consistent with our in vitro binding studies, without exposing the DEE endocytic signal (40, 56). Thus, it appears that obscurin-kin1–mediated phosphorylation of N-cadherin at Ser-788, which results in reduced binding to p120-catenin, is not involved in N-cadherin internalization and degradation.

In addition to regulating the trafficking and stability of N-cadherin at the plasma membrane, cadherin-uncoupled, cytoplasmic p120-catenin potently inhibits RhoA activity, either directly via binding or indirectly via Rac1 activation, impacting its ability to modulate the (re)organization of the cortical cytoskeleton (39, 57, 58). Importantly, the dynamic binding of p120-catenin to cadherins appears to be regulated via phosphorylation (59). Consistent with this, phosphorylation of E-cadherin at Tyr-755/756 or VE-cadherin at Tyr-658 disrupts p120-catenin binding. We therefore postulate that obscurin-kin1–mediated phosphorylation of N-cadherin at Ser-788 results in reduced N-cadherin/p120-catenin binding, which in turn frees up p120-catenin to negatively regulate RhoA activity. This notion would be consistent with elevated RhoA activity in the absence of N-cadherin phosphorylation at Ser-788, which is in agreement with our findings following overexpression of phosphoablated N-cadherin. More importantly, in accordance with our studies indicating enhanced mechanochemical coupling of embryonic cardiomyocytes following overexpression of obscurin-kin1 or phosphomimic N-cadherin, reduced RhoA activity has been implicated in promoting cell adhesion (39, 58, 60).

Given the reported involvement of the *OBSCN* gene in different forms of cardiomyopathies (19) along with the elevation of obscurins in preclinical models of myocardial hypertrophy and ventricular tachycardia (17, 61, 62), our findings demonstrating upregulation of the obscurin-B/phospho-N-cadherin axis in end-stage DCM may be of high pathophysiological relevance. As such, it is plausible that upregulation of the obscurin-B/phospho-N-cadherin axis may underlie enhanced mechanical coupling and thus elevated stiffness consistent with the DCM pathology as well as increased electrical coupling leading to changes in cardiomyocyte conductance and development of arrhythmia. Consistent with this, 7 out of the 9 DCM patients we evaluated presented with atrial fibrillation, while 2 also exhibited ventricular tachycardia (Table 1). It is important to note that of the approximately 20 known *OBSCN* variants linked to the development of heart disease, 3 immediately flank kin1 and are associated with the development of left ventricular noncompaction cardiomyopathy or DCM, while 1 resides within the kin1 catalytic domain, possibly affecting its enzymatic activity and/or substrate specificity and is associated with the development of DCM.

Taken together, our studies uncover what we believe is a previously unknown axis mediating cardiomyocyte coupling that involves the regulation of N-cadherin via obscurin-kin1–mediated phosphorylation at Ser-788. Given the nearly ubiquitous expression of both obscurin and N-cadherin, our findings may have broad applicability and impact in identifying and characterizing this signaling axis that may contribute to the regulation of cell coupling in diverse tissues and organs.

## Methods

### Primary antibodies.

All primary antibodies used in the study are included in Supplemental Methods.

### Expression constructs and recombinant proteins.

A description of the source or generation of constructs and recombinant proteins is provided in Supplemental Methods.

### Preparation and culturing of ERVMs.

ERVMs were isolated from hearts of E21 Sprague-Dawley rats, as previously described (4). A description of the procedure is included in Supplemental Methods.

### Ca^2+^ switch assay.

ERVMs and HEK293 cells were plated on laminin-coted (20 μg/mL) coverslips and cultured overnight in DMEM-F12 media containing 5% FBS, 100 U/mL penicillin-streptomycin and DMEM media containing 10% FBS, 100 U/mL penicillin-streptomycin, respectively, in the presence of 1.8 mM CaCl_2_ (Thermo Fisher Scientific) to allow cell attachment before switching to medium lacking Ca^2+^. Following incubation for 4 hours in Ca^2+^-depleted media, cells were switched back to Ca^2+^-containing media for 1 hour, and subsequently processed for immunofluorescent staining (please see below) followed by confocal imaging.

### Transfection of ERVMs followed by immunofluorescent staining and confocal evaluation.

ERVMs were plated on laminin-coated (20 μg/mL) coverslips and allowed to adhere overnight before they were transfected with 1 μg/mL control pEGFPN1 or pEGFPN1-Kin1-CA plasmids using the TransIT-LT1 transfection reagent (Mirus Bio LLC) according to the manufacturer’s instructions. Seventy-two hours after transfection, ERVM cultures were fixed with ice-cold 100% methanol for 30 minutes at –20°C, permeabilized with 0.1% Triton X-100 for 5 minutes, blocked with 5% goat serum for 1 hour, and incubated with the indicated primary antibodies overnight at 4°C. Following extensive washes with PBS, samples were incubated with the appropriate secondary antibodies conjugated with Alexa Fluor dyes (Thermo Fisher Scientific) for 1 hour at room temperature and mounted with Prolong antifade mounting medium (Thermo Fisher Scientific). Samples were imaged under confocal optics (Zeiss LSM510 META NLO or LSM DUO) with an ×63 objective.

### Transfection of HEK293 cells and cell fractionation.

HEK293 cells were plated on 6-well plates and following establishment of a confluent monolayer they were transfected with 2.5 μg of control pEGFPN1 or pEGFPN1-Kin1-CA plasmid using Lipofectamine 3000 (Thermo Fisher Scientific). Cell fractionation was performed 72 hours after transfection using the MEM-PER Plus membrane protein extraction kit (Thermo Fisher Scientific) to separate the membrane and cytosolic fractions, as described by the manufacturer. Equivalent amounts of cytosolic and membrane protein fractions were resolved by Novex NuPAGE SDS-PAGE (Thermo Fisher Scientific), transferred to nitrocellulose membranes, and probed with the indicated primary antibodies followed by the appropriate horse radish peroxidase–conjugated (HRP-conjugated) secondary antibodies (i.e., goat anti–rabbit IgG, 1:3,000 dilution; Cell Signaling Technology, catalog 7074). Immunoreactive bands were visualized with SignalFire ECL reagent (Cell Signaling Technology) in an Amersham Imager 680 (Amersham Biosciences).

### Dispase assay.

ERVM cultures were transiently transfected as described above with the following plasmids: pEGFP-N1, pEGFP-N1-Kin1-CA, pEGFP-N1-N-cad-WT, pEGFP-N1-N-cad-S788E, and pEGFP-N1-N-cad-S788A. Seventy-two hours after transfection, ERVMs were sorted for GFP fluorescence via flow cytometry (FACSAria II, BD Biosciences). GFP-positive cells were replated in a 48-well plates precoated with Geltrex (0.035 mg/mL; Life Technologies), and subjected to Dispase assay, as in Ackermann et al. (4); a description of the Dispase assay is included in Supplemental Methods.

### Dye transfer assay.

ERVM cultures transfected with pEGFP-N1, pEGFP-N1-Kin1-CA, pEGFP-N1-N-cad-WT, pEGFP-N1-N-cad-S788E, and pEGFP-N1-N-cad-S788A were subjected to dye transfer assays, as described previously (63, 64) with minor modifications; a description is provided in Supplemental Methods.

### Expression and purification of His-tagged obscurin-kin1 from insect cells.

A detailed description of the expression and purification of His-tagged obscurin-kin1 is provided in Supplemental Methods.

### In vitro kinase assay and MS/MS.

6×His-Kin1-CA purified protein (1 μg) or negative control was incubated with 1 μg of His-Ncad_746–906_ including the cytoplasmic domain of N-cadherin or His-Ncad_786–880_ including part of the cytoplasmic domain of N-cadherin enriched in potential Ser/Thr phospho-sites, in kinase buffer containing 10 mM Na_2_HPO_4_, 10 mM MgCl_2_, 200 μM ATP, 1 mM CaCl_2_, 1 mM DTT, and 100 nM okadaic acid, in the presence of Halt protease and phosphatase inhibitors (Thermo Fisher Scientific) at 4°C overnight. The reaction mixtures were subsequently subjected to MS/MS (Johns Hopkins School of Medicine, Mass Spectrometry Core) to examine the presence of phosphorylation site(s). A description of the MS/MS is included in Supplemental Methods. Phospho-Ser-788 was identified 1 and 6 times in His-Ncad_746–906_ and His-Ncad_786–880_, respectively, with 100% probability. Data are available via ProteomeXchange (https://www.proteomexchange.org/) with identifier PXD031003.

### Determination of V_max_, K_M_, and K_cat_ of the obscurin-kin1/N-cadherin-Ser-788 phosphorylation reaction.

The Universal Fluorometric Nonradioactive Assay kit (Abcam, ab138879) was used to detect and measure the in vitro kinase activity of His-Kin1-CA. Briefly, the activity of His-Kin1-CA was assessed by measuring ADP formation, which is directly proportional to the enzyme’s phosphotransferase activity. Fluorescence intensity was measured at varying substrate concentrations (70–2900 nM) with a fluorescence plate reader (Synergy HTX, BioTek) at excitation/emission of 540/590 nm. Substrates, including GST-Ncad_746–906_-WT, GST-Ncad_746–906_-SA, and GST, were incubated with a constant amount of His-Kin1-CA, set at 26 nM, and 100 μM ATP in a 96-well plate in the dark for 1 hour at 25°C. At the end of the incubation period, 20 μL of ADP sensor buffer and 10 μL of fluorescent ADP sensor were added to each reaction. Relative fluorescence units (RFU) at each substrate concentration were measured in duplicate and converted to total nanomoles of ADP produced using an ADP standard curve. The amount of ADP formed per minute at each substrate concentration was calculated by dividing the total nanomoles of ADP formed by the reaction time (i.e., 60 minutes). Nanomoles of ADP formed per minutes from *n* = 3 independent experiments performed in duplicate were fitted using GraphPad Prism nonlinear regression software for Michaelis-Menten kinetics to calculate the apparent *V_max_* and *K_M_*, while the apparent *K_cat_* was obtained by dividing the fitted *V_max_* by the total nanomoles of the enzyme. The 3 experiments are independently presented in Figure 3A and Supplemental Figure 2 (instead of being averaged), due to the generation and use of different standard curves; all 3 experiments yielded similar catalytic turnover rates.

### Preparation of protein lysates from embryonic and adult rat hearts.

E21 and 3-month-old adult rat hearts were harvested and homogenized in ice-cold lysis buffer containing 10 mM Na_3_PO_4_ (pH 7.2), 2 mM EDTA, 10 mM NaN_3_, 120 mM NaCl, and 1% NP-40 supplemented with Halt protease and phosphatase inhibitors (Thermo Fisher Scientific), using a microbeads homogenizer (TissueLyser LT). Following tissue homogenization, samples were incubated at 4°C for approximately 60 minutes using gentle rotation prior to centrifugation at 13,400*g* and 4°C for 20 minutes. Protein concentration was determined using the bicinchoninic acid (BCA) protein assay (BCA protein assay kit, Thermo Fisher Scientific). Twenty milligrams of protein lysates from embryonic and adult hearts was separated by Novex NuPAGE SDS-PAGE (Thermo Fisher Scientific), transferred to nitrocellulose membranes, and probed with the indicated primary antibodies followed by the appropriate HRP-conjugated secondary antibodies (i.e., goat anti–rabbit IgG, 1:3,000 dilution; Cell Signaling Technology, catalog 7074).

### Immunofluorescent staining of ERVM cultures and adult mouse hearts.

ERVMs isolated from *n* = 12 E21 Sprague-Dawley rats were plated on ibidi-coated 96-well plates (μ-Plate 96-Well Black; ibidi GmbH) and allowed to adhere overnight. Three days after plating, ERVM cultures were fixed with 4% paraformaldehyde (PFA) in PBS for 30 minutes, permeabilized with 0.1% Triton X-100 for 10 minutes, blocked with 1 mg/mL BSA, 1 mM NaN_3_, and 50 mM glycine in PBS for 1 hour, and incubated with N-Cad-phospho-Ser-788 (20 μg/mL) and total N-cadherin (dilution 1:200; Thermo Fisher Scientific, clone 3B9) antibodies. Three adult mice (1, 3, and 6 months old) were perfused with 4% PFA under anesthesia. Hearts were excised and embedded in OCT Embedding Compound (Tissue-Tek, Sakura) and gradually frozen using 2-methylbutane at –60°C. Cardiac sections (~10 μm) were obtained with a Micron HM550 cryostat (Thermo Fisher Scientific), permeabilized with 0.1% Triton X-100 for 25 minutes, blocked with 1 mg/mL BSA, 1 mM NaN_3_, and 50 mM glycine in PBS for 1 hour, and incubated overnight with N-Cad-phospho-Ser-788 (40 μg/mL) and total N-cadherin (dilution 1:200; Thermo Fisher Scientific, clone 3B9) antibodies. Following extensive washes with PBS, ERVM cultures and adult cardiac sections were incubated with the appropriate secondary antibodies conjugated to Alexa Fluor 488 and Alexa Flour 647 for 2 hours at room temperature and then counterstained with Hoechst (1:400; Invitrogen). Adult cardiac sections were mounted with ProLong Diamond Antifade Mountant (Invitrogen). Specimens were analyzed under a Nikon Eclipse Ti2 spinning disk confocal microscope equipped with an ×60, 1.49 numerical aperture oil immersion TIRF objective using dual laser settings at 525 nm and 665 emission wavelengths with 500 ms and 200 ms exposure time, respectively.

### Insulin treatment of ERVM cultures.

ERVM cultures were serum starved overnight prior to treatment with 10 nM insulin (Sigma-Aldrich, 10516) for the indicated time points (i.e., 0, 10, 30, 60, 180, and 30 minutes), subsequently washed with ice-cold PBS (twice), and harvested in ice-cold lysis buffer containing 10 mM Na_3_PO_4_ (pH 7.2), 2 mM EDTA, 10 mM NaN_3_, 120 mM NaCl, and 1% NP-40 supplemented with Halt protease and phosphatase inhibitors (Thermo Fisher Scientific). Following incubation on ice for 60 minutes and gentle pipetting, cell lysates were obtained by centrifugation at 13,400*g* at 4°C for 20 minutes. Thirty micrograms of protein lysate from each time point was evaluated via immunoblotting using the indicated primary antibodies followed by the appropriate alkaline phosphatase–conjugated (AP-conjugated) secondary antibodies (i.e., goat anti–mouse IgG, 1:3,000 dilution; Sigma-Aldrich, catalog A3688 and goat anti–rabbit IgG, 1:3,000 dilution; Jackson Immunoresearch, catalog AB_2337947), while immunoreactive bands were visualized by chemiluminescence (NovaBright, Thermo Fisher Scientific).

### GST pull-down assays.

Equivalent amounts of GST, GST-Ncad_746–906_-WT, GST-Ncad_746–906_-S788E, and GST-Ncad_746–906_-S788A were immobilized on glutathione sepharose beads via their GST tag and incubated with 1 mg of 3-month-old adult mouse heart lysates prepared in lysis buffer containing 10 mM Na_3_PO_4_, pH 7.2, 10 mM NaN_3_, 120 mM NaCl, 1 mM CaCl_2_, and 1% NP-40 supplemented with a cOmplete protease inhibitor tablet (Roche Applied Science) at 4°C overnight. Following extensive washes with lysis buffer, complexes were eluted with 2× SDS loading buffer, boiled for 10 minutes, resolved by SDS-PAGE, and analyzed via immunoblotting using the indicated primary antibodies followed by the appropriate AP-conjugated secondary antibodies (i.e., goat anti-mouse IgG, 1:3,000 dilution; Sigma-Aldrich, catalog A3688 and goat anti–rabbit IgG, 1:3,000; Jackson Immunoresearch, catalog AB_2337947). Immunoreactive bands were visualized by chemiluminescence (NovaBright, Thermo Fisher Scientific). Binding of p120-catenin or β-catenin to GST-Ncad_746–906_-S788E and GST-Ncad_746–906_-S788A is expressed relative to GST-Ncad_746–906_-WT, which was arbitrarily set to 1.

### ITC.

The binding interaction of ligands GST-Ncad_476–906_-WT and GST-Ncad_476–906_-SE to sample macromolecule MBP-p120-catenin_311–747_ was monitored using a VP-ITC titration microcalorimeter (MicroCal Inc.) at 37°C. For all experiments, ligands and sample macromolecule were dialyzed into PBS pH 7.4 (Quality Biological), degassed under vacuum, and equilibrated at 37°C prior to titration. Ligand solutions varied from 80–100 μM of total protein, while the ITC sample chamber (1.8 mL volume) contained 10 μM MBP-P120-catenin_311–747_. Three sets of control experiments were performed: in the first, 100 μM of each ligand was injected into PBS buffer; in the second, 100 μM of each ligand was injected into PBS containing 10 μM MBP; and in the third, 100 μM GST was titrated into PBS containing 10 μM MBP-p120-catenin_311–747_. Heat change from control titrations was subtracted from experimental titrations. Three different recombinant protein preparations were used for each of the 3 experimental replicates, with all showing similar results. Data were fit to a 1-site binding model using Origin (MicroCal Inc.) to obtain *K_D_* values. The *K_D_* values from all 3 experimental replicates were averaged and the SEM was calculated. The number of binding sites for GST-Ncad_476–906_-SE titrations was manually fixed to 1 for model fitting due to the considerably weak interaction.

### Computational modeling.

Molecular dynamics simulations using the mouse N-cadherin/p120 complex model (based on the human E-cadherin/p120-catenin structure; PDB accession number 3L6X) were run on WT, the S787E phosphomimic variant, and the phospho-S788 form of N-cadherin, introduced by the swap command in YASARA (41, 65). These simulations were run at 310°K, 150 mM NaCl, pH 7.4 using the Amber ff14 forcefield in a simulation cell with periodic boundaries for 100 ns. Simulations were run with a time step of 1.25 fs with the temperature adjusted using a Berendsen thermostat, as described in Krieger et al. (65). Both simulations stabilized within 5 ns as judged by RMSD, and analyses were performed in 100-ps intervals from 5 to 100 ns using YASARA macros.

### Immunoblotting and immunofluorescence analysis of HEK293 cells ectopically expressing GFP-N-cadherin variants.

HEK293 cells were plated on glass coverslips precoated with Geltrex (0.035 mg/mL, Life Technologies) in 35-mm dishes and allowed to reach 80%–90% confluence, at which time point they were transfected with 1 μg/μL control pEGFP-N1, pEGFP-N1-N-cad-WT, pEGFP-N1-N-cad-S788E, and pEGFP-N1-N-cad-S788A plasmids using the TransIT-LTI transfection reagent (Mirus Bio LLC). Forty-eight hours after transfection, HEK293 cultures were processed for either immunoblotting or immunofluorescence analysis. Specifically, protein lysates were prepared using RIPA buffer (Thermo Fisher Scientific, 89900) containing protease/phosphatase inhibitors (Thermo Fisher Scientific, 78446). Immunoblotting was performed as described above, while detection of immunoreactive bands was performed with the ECL detection system (SignalFire ECL reagent, Cell Signaling Technology, 6883). Conversely, cells were fixed with ice-cold 100% methanol for 30 minutes at –20°C, permeabilized with 0.1% Triton X-100 for 5 minutes, blocked with 5% goat serum for 1 hour, and incubated with the indicated primary antibodies overnight at 4°C. Following extensive washes with PBS, samples were incubated with the appropriate secondary antibodies conjugated with Alexa Fluor dyes (Thermo Fisher Scientific) for 1 hour at room temperature and mounted with Prolong antifade mounting medium (Thermo Fisher Scientific). Samples were imaged under confocal optics (Zeiss LSM510 META NLO or LSM DUO) with an ×63 objective.

### RhoA activity measurements using G-LISA assay.

The levels of active RhoA (GTP-RhoA) were evaluated using the G-LISA RhoA luminescence activation assay kit (Cytoskeleton, BK121) following the manufacturer’s instructions. In brief, following lysis of HEK293 cells transfected with 1 μg/μL of control pEGFP-N1, pEGFP-N1-N-cad-WT, pEGFP-N1-N-cad-S788E, and pEGFP-N1-N-cad-S788A plasmids, 50 μL of each sample and the appropriate negative (blank) and positive controls were assayed in 96-well G-LISA plates, while the signal was measured using a microplate luminometer (Synergy, Agilent). Moreover, the levels of total RhoA were evaluated via immunoblotting analysis as described above using 20 mg of protein lysates prepared in RIPA buffer (Thermo Fisher Scientific, 89900) and the ECL detection system (reagent, Cell Signaling Technology, 6883).

### RNA isolation and RT-PCR.

A description of the RNA isolation method and RT-PCR is provided in Supplemental Methods.

### Preparation of protein lysates from control and heart failure human samples.

Human donor control and DCM heart failure samples were obtained from the Sydney Heart Bank, Australia in collaboration with St. Vincent’s Hospital, Sydney. Samples included 9 end-stage DCM LVs consisting of 6 males and 3 females ranging in age between 31 and 64 years old, and 6 age-matched donor LVs consisting of 5 males and 1 female, ranging in age between 29 and 63 years old (Table 1).

Protein lysates from control and heart failure LV samples were prepared as described previously (17). In brief, samples were ground to powder in liquid nitrogen with a mortar and pestle. The ground tissue was kept at –20°C for 20 minutes, followed by solubilization in lysis buffer containing 8 M urea, 2 M thiourea, 3% (w/v) SDS, 0.05 M Tris-HCl (pH 6.8), 0.075 M dithiothreitol (DTT), 0.03% (w/v) bromophenol blue, and 10% (w/v) glycerol, supplemented with Halt protease and phosphatase inhibitors (Thermo Fisher Scientific) in a 60°C water bath. Following centrifugation, aliquoted protein lysates were flash frozen in liquid nitrogen and immediately stored at –80°C. Given that the presence of urea/thiourea does not allow determination of protein concentration, starting from equal amounts of control and experimental tissue enables equivalent loading among samples (17). Accordingly, equal volumes of control and heart failure protein lysates were heated at 55°C for 5 minutes, resolved by Novex NuPAGE SDS-PAGE (Thermo Fisher Scientific), transferred to nitrocellulose membranes, and probed with the indicated primary antibodies followed by the appropriate HRP-conjugated secondary antibody (i.e., goat anti–rabbit IgG, 1:3,000 dilution; Cell Signaling Technology, 7074). Immunoreactive bands were visualized by ECL chemiluminescence (SignalFire ECL reagent, Cell Signaling Technology, 6883).

### Presentation of Coomassie Blue gels and immunoblots.

All Coomassie Blue gels and immunoblots are shown in grayscale mode for consistency of presentation. Given the limited availability/amounts of some samples, horizontal strips from the same blot were cut across at the appropriate molecular weight and probed with the relevant antibodies to ensure that the expression levels of proteins of interest were evaluated in the same lysates.

### Statistics.

Statistical tests, sample numbers, number of biological/technical repeats, and *P* values are provided in figure legends; values are expressed as mean ± SEM. A *P* value of less than 0.05 was considered significant. Densitometric evaluation of immunoreactive bands was performed with ImageJ software (NIH).

### Study approval.

All animal work was conducted under a protocol approved by the Institutional Animal Care and Use Committee (IACUC) of the University of Maryland School of Medicine. Human heart tissue used in this study was collected with the informed and written consent of transplant patients or from the families of organ donors of nonfailing hearts in accordance with the principles in the Declaration of Helsinki. Ethical approval was provided by the Human Research Ethics Committees at the University of Sydney (2016/7326) and St. Vincent’s Hospital (H03/118). Material transfer agreement was executed between the University of Sydney and the University of Maryland Baltimore, School of Medicine (reference no. CT14876).

### Data availability.

Raw blots, gels, and images are included in the supplemental material pdf file, with areas shown in figures marked with white dashed or red solid boxes. In addition, values for all data points shown in graphs are included in the Supporting Data Values xls file.

## Author contributions

LW, PT, and RG conceptualized the study, performed experiments, analyzed data, validated results, and wrote and edited the manuscript. SC performed experiments, analyzed data, validated results, and edited the manuscript. AL and CDR provided resources and edited the manuscript. NTW performed modeling, analyzed data, wrote and edited the manuscript, and acquired funding. AKK conceptualized the study, wrote and edited the manuscript, supervised the project, provided project administration, and acquired funding.

## Supplementary Material

Supplemental data

Supporting data values

## Figures and Tables

**Table 1:**
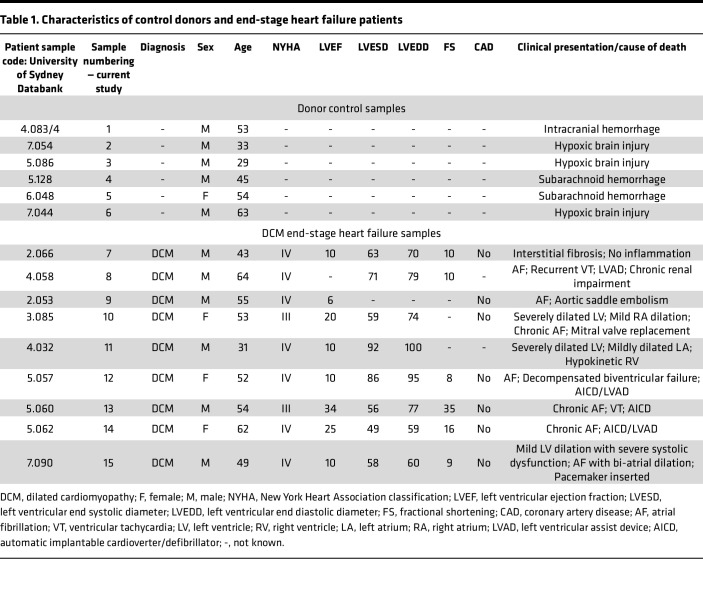
Characteristics of control donors and end-stage heart failure patients

## Version 1 12/21/2023

In-Press Preview

## Version 2 02/08/2024

Electronic publication
