# Supplementary material for: Essential role of obscurin kinase-1 in cardiomyocyte coupling via N-cadherin phosphorylation
Source: JCI Insight. 2024 Feb 8;9(3):e162178. doi: 10.1172/jci.insight.162178 (PMC10967385; doi:10.1172/jci.insight.162178)

Supplemental Methods

Primary Antibodies

The N-Cad-pS788 and N-Cad-S788 antibodies were generated in rabbits by injection of the Keyhole Limpet Hemocyanin (KLH)-conjugated phosphopeptide NH_2-DQDYDL(pS)QLQQPD-COOH (aa 782-794; accession #: P19022, Uniprot: https://www.uniprot.org/uniprot/P19022). Anti-serum was sequentially purified over columns conjugated with the above phosphopeptide and the corresponding depleting peptide (NH_2-DQDYDLSQLQQPD-COOH) to collect N-Cad-pS788 and N-Cad-S788 antibodies (Thermo Fisher Scientific, Rockford, IL). The specificity of the obtained antibody lots was tested by ELISA against both peptides, which confirmed that the N-Cad-pS788 antibody is phospho-specific since it did not react with the depleting peptide. The N-Cad-pS788 antibody was used at 600 ng/ml and the N-Cad-S788 antibody was used at 300 ng/ml in subsequent immunoblotting experiments. Moreover, the obscurin-kinase 1 (obsc-Kin1) antibody was generated in rabbits by injection of a recombinant protein containing the catalytic portion of kin1 tagged to 6x-His (6xHis-ObscKin1-CA, ~34 kDa; aa 7401-7669; accession #: A2AAJ9, Uniprot: http://www.uniprot.org/uniprot/A2AAJ9; Thermo Fisher Scientific). Anti-serum was purified over a cyanogen bromide (CNBr) column coupled to 6xHis-ObscKin1-CA, as in (1), and the obtained obsc-Kin1 antibody was used at 1 μg/ml in the relevant immunoblotting experiments.

The following commercial and custom-made antibodies were also used in immunoblotting experiments at the indicated concentrations or dilutions: anti-GFP (A-11122; dilution: 1:2000; Thermo Fisher Scientific), anti-N-Cadherin (610920; dilution 1:2000; BD Biosciences, San Jose, CA), anti-β-catenin (clone 6B3; #9582; dilution: 1:1,000; Cell Signaling Technology, Danvers,

MA), anti-delta1-catenin/p120-catenin (ab92514; dilution: 1:1000; Abcam, Cambridge, MA), and anti-Na/K ATPase-α1 (MA3-929; dilution: 1:1000; Thermo Fisher Scientific). Moreover, anti-RhoA (#2117; dilution: 1:1,000; Cell Signaling Technology) and anti-RhoA (#GL01A; dilution: 1:250; Cytoskeleton, Denver, CO) antibodies were used for total and active RhoA expression in immunoblotting and G protein-linked (G-LISA) assays, respectively. Lastly, the following antibodies were used in immunofluorescence experiments: anti-obscB (300 ng/ml), which recognizes epitopes within Ig69 and Fn-III70 (1), anti-N-Cad-pS788 (20 or 40 μg/ml), anti-N-Cadherin (610920; dilution: 1:200; BD Biosciences) and anti-N-cadherin (3B9, dilution 1:200, Thermo Fisher Scientific).

Expression constructs and recombinant proteins

The following Green Fluorescent Protein (GFP) constructs were generated or purchased: the catalytic domain of the mouse obscurin-kin 1 (aa 7401-7669, accession #: A2AAJ9, Uniprot: https://www.uniprot.org/uniport/A2AAJ9) was subcloned into the pEGFP-N1 vector (GenBank accession #: U55761, Clontech, Mountain View, CA) at BamH1/Xho1 restriction sites (1); the mouse full length WT pEGFP-N1-N-cadherin plasmid was commercially obtained [catalogue #: 18870; Addgene, Watertown, MA; deposited by Dr. Vasioukhin's group (2)], and the mouse pEGFP-N1-N-cadherin-S788E and pEGFP-N1-N-cadherin-S788A constructs were generated with the QuickChange II site-directed mutagenesis kit (Agilent, Santa Clara, CA), according to the manufacturer's instructions. Moreover, the cytoplasmic domain of mouse N-cadherin (aa 747-906, accession # P15116, Uniprot: https://www.uniprot.org/uniprot/P15116; $Ncad_{476-906}$) was subcloned into the pGEX4T1 vector at BamH1/Xho1 sites (GE Healthcare, Chicago, IL), while GST-tagged phosphomimic ($Ncad_{746-906}$-S788E) and phosphoablated ($Ncad_{746-906}$-S788A)

constructs containing the cytoplasmic domain of N-cadherin were generated with the QuickChange II site-directed mutagenesis kit (#200521, Agilent), following the manufacturer's instructions. Protein expression was induced by 0.5 mM IPTG for 3-4 hours at 37°C, and recombinant proteins were affinity-purified using glutathione-sepharose beads (GE Healthcare Life Sciences, Marlborough, MA). A shorter portion of the cytoplasmic domain of N-cadherin (aa 786-880, accession # P15116, Uniprot: https://www.uniprot.org/uniprot/P15116; $Ncad_{786-880}$) enriched in potential Ser/Thr phospho-sites was subcloned into the pET21a vector (Addgene) at NdeI/XhoI sites. Protein expression was induced by 1 mM IPTG for 3-4 hours at 37°C, and the recombinant protein was affinity-purified using the Ni-NTA His Bind Superflow resin (EMD Millipore, Billerica, MA). Moreover, a fragment of mouse p120-catenin (aa 311-747, accession # P30999, Uniprot: https://www.uniprot.org/uniprot/P30999) containing armadillo domains 1-8 was cloned into the pMAL-c2E vector at EcoRI/HindIII sites. Protein expression was induced by 1 mM IPTG overnight at 18°C, and the recombinant protein was affinity-purified using the pMAL protein fusion and purification system kit (#E8200S, New England Biolabs, Ipswich, MA). The authenticity of all constructs was verified by sequencing before proceeding to further experimentation. The primer sets used for the generation of the indicated constructs are provided in **Supplemental Table 1**.

Preparation and culturing of embryonic rat ventricular myocytes (ERVM)

ERVM were obtained from hearts of embryonic day 21 (E21) Sprague-Dawley rats, as previously reported (3); of note, ERVM form cell junctions, which are considered "primordial ICDs" (4), allowing the study of de novo formation of cell junctions rather than disassembly and reassembly of ICDs that takes place in adult cardiomyocyte cultures. In brief, euthanasia of

pregnant dams via carbon dioxide (CO_2) asphyxiation was confirmed by cervical dislocation, in accordance with the Guide for the Care and Use of Laboratory Animals of the National Institutes of Health. E21 embryos were decapitated on ice, the hearts were excised, and the atria were removed. Ventricles were rinsed in PBS, followed by 5-6 rounds of successive digestions of 10-15 min each with a mixture of collagenase II (Worthington Biomedical Corporation, Lakewood, NJ) and pancreatin (Sigma-Aldrich, St. Louis, MO) dissolved in 116 mM NaCl, 20 mM HEPES, 1 mM NaH_2PO_4, 5.5 mM Glucose, 5.4 mM KCl, 0.8 mM $MgSO_4$, and phenol red, pH 7.4. The supernatants (from the series of successive digestions) were removed from the tissue "chunk" and each placed in a 15 ml conical tube containing 10% heat-inactivated horse serum (Thermo Fisher Scientific). After centrifugation, cardiomyocyte fractions were resuspended in 100% horse serum, combined, and filtered through a sterile cell strainer with mesh size of 70 μm (Thermo Fisher Scientific). The filtrate was subsequently centrifuged at 2,000 rpm for 5 min, and the cell pellet was resuspended in the proper volume of plating medium containing DMEM-F12 (Thermo Fisher Scientific), 10% heat-inactivated FBS (Thermo Fisher Scientific), 200 U/ml penicillin and 200 U/ml streptomycin (Thermo Fisher Scientific) to obtain a final cell density in the range of 0.5-1 x 10^6 cells/ml. Twenty-four hours after plating, the plating medium was replaced with growth medium containing DMEM-F12, 5% FBS, 100 U/ml penicillin and 100 U/ml streptomycin.

Dispase assay

Confluent ERVM monolayers transfected with the indicated plasmids were gently washed with PBS, followed by treatment with 10 μM blebbistatin for 30 min to inhibit contraction, and then incubated with 2.5 U/ml dispase at 37°C/5%CO_2 for 4-6 hr. The detached cell monolayer was subsequently subjected to mechanical stress achieved using an orbital shaker (70 rpm) for 30 min.

Fragmentation of monolayers was imaged with a HAMAMATSU Digital Camera (ORCA-Flash4.0LT; MODEL: C11440-42U; S/No. 000452; HAMAMATSU Photonics) connected to a Leica MZ10F Stereo Microscope (MODEL MSV269; S/NO 5828800), and evaluation of cell fragment numbers was performed with ImageJ software.

Dye transfer assay

Seventy-two hr post-transfection, GFP-positive ERVM were randomly assigned to either donor or recipient groups. Donor cells (1 x 10^6 cells/ml) were labeled with 10 μM of the GJ transferrable calcein deep red acetate dye (AAT Bioquest, Sunnyvale, CA), while recipient cells (1 x 10^6 cells/ml) were labeled with the GJ non-transferable CytoTell Red 590 dye (1:500x dilution; AAT Bioquest) in a 37°C/5% CO_2 incubator for 1 hr. Following labeling, cells were gently washed three times with PBS, and subsequently mixed and incubated at 37°C/5% CO_2 for 2 hr. Evidence of calcein deep red acetate transfer from donor to recipient cells was evaluated by flow cytometry (BD LSR II; BD Biosciences). The rate of dye transfer was calculated with the following formula: number of cells positive for both calcein deep red and CytoTell Red 590/number of cells positive for CytoTell Red 590 x 100% (5).

Expression and purification of His-tagged obscurin kinase-1 from insect cells

The catalytic domain of the mouse obscurin-kin1 (aa 7401-7669, accession # A2AAJ9, Uniprot: https://www.uniprot.org/uniprot/A2AAJ9) conjugated to the 6xHis tag (6xHis-ObscKin1-CA) (1) was subcloned into the pUC57 baculovirus expression vector (GenScript, Piscataway, NJ). Following sequence verification, the recombinant plasmid was transfected into DH10Bac competent cells for production of recombinant bacmid (rbacmid), which was transfected

into Sf9 insect cells using Cellfectin II (Thermo Fisher Scientific). Sf9 cells were subsequently incubated in Sf-900 II serum free media for 5-7 days at 27°C. At the end of the incubation period, Sf9 cells were centrifuged, and the supernatant was collected and designated as baculovirus P1 virus stock (GenScript). The P1 viral stock was used to infect a new batch of Sf9 cells to scale-up production and obtain viral stock P2. 6xHis-ObscKin1-CA recombinant protein was purified from viral stock P2 72 hours post-infection by pelleting, harvesting, and lysing the Sf9 cells in a buffer containing 50 mM Na_3PO_4, 300 mM NaCl, 20 mM imidazole, 8 M Urea, and 0.5 mM DTT supplemented with a cOmplete^{TM} protease inhibitor cocktail (Roche, Mannheim, Germany), pH 7.4. The clarified Sf9 cell lysate was subsequently affinity-purified under denaturing conditions through a His60 Ni Superflow Resin (Clontech Laboratories, Mountain View, CA), according to the manufacturer's instructions. In brief, the Sf9 cell lysate was added to His60 Ni superflow resin column and gently rocked at 4°C for 1 hour to allow binding of the 6xHis-ObscKin1-CA protein. Following extensive washes with a buffer containing 50 mM Na_3PO_4, 300 mM NaCl, 60 mM imidazole, 8 M Urea, the recombinant protein was eluted by adding successively elution buffer A (5 column volumes): 50 mM Na_3PO_4, 300 mM NaCl, 150 mM imidazole, 8 M Urea, and buffer B (5 column volumes): 50 mM Na_3PO_4, 300 mM NaCl, 300 mM imidazole, 8 M Urea. A total of 10 eluant fractions (5 fractions from buffer A and another 5 from buffer B) were collected, which were subjected to dialysis in PBS, followed by buffer exchange and concentration via Amicon centricon (EMD Millipore, Burlington, MA) in 50 mM Tris-HCl, 150 mM NaCl, and 1 mM TCEP-HCl in the presence of cOmplete^{TM} protease inhibitors. Non-infected Sf9 cells went through the same purification procedure as 6xHis-ObscKin1-CA expressing cells, and the final product served as negative control in subsequent experiments.

Tandem Mass Spectrometry

The in vitro kinase reaction mixtures were subjected to in-solution digestion as described in (6). Following pH adjustment to 8 with triethylammonium bicarbonate (TEAB) buffer, protein mixtures were reduced with 3 mg/ml DTT for 1 hr at 56°C, alkylated with 15 mg/ml iodoacetomide for 45 min at room temperature in the dark, and digested with 12.5 ng/µl trypsin/Lys-C mixture (Promega, Madison, WI) at 37°C overnight. Post-digestion, the peptides were acidified and desalted on Oasis µ-HLB plates (Waters Corporation, Milford, MA), and eluted with 60% acetonitrile (ACN) and 1% trifluoroacetic acid (TFA). The eluted peptides from each reaction mix were split into two halves and dried. One half was directly used for liquid chromatography tandem-mass spectrometry (LC-MS/MS) and the other half was enriched for phosphopeptides using titanium dioxide (TiO_2). Not enriched peptides were analyzed on an QExactive HF (Thermo Fisher Scientific) interfaced with a nano-Acquity LC system from Waters by reversed-phase chromatography using a 2%–90% acetonitrile in 0.1% formic acid gradient over 76 min at 300 nl/min on a 75 µm x 150 mm ProntoSIL-120-5-C18 H column 5 µm, 120Å (BISCHOFF; http://www.bischoff-chrom.com/hplc-prontosil-c18-h-c18-phasen.html). Eluting peptides were sprayed into the mass spectrometer through a 1 µm emitter tip (New Objective) at 2.2 kV. Survey scans (MS) of precursor ions were acquired from 350-1800 m/z at 120,000 resolution for 200 m/z, 3xe6 automatic gain control (AGC), 100 ms maximum injection time (IT). Precursor ions were individually isolated within m/z 1.6Da with 0.5Da offset by data dependent monitoring and 15s dynamic exclusion, and fragmented (MS/MS) using an HCD activation collision energy 28. MS/MS spectra were acquired using a 1e5 automatic gain control (AGC), 200 ms maximum injection time (IT) at 30,000 resolution at 200Da and a 271.1012 lock mass. Phosphopeptides, enriched by binding to TiO_2 beads, were similarity analyzed except for the following changes.

Phosphoenriched peptides were analyzed on an QExactive Plus (Thermo Fisher Scientific) interfaced with an Easy-nLC1100 UPLC. Precursor ions were acquired at 70,000 resolution for 200 m/z and MS/MS spectra were acquired at 35,000 resolution at 200Da using a 271.1012 lock mass. Isotopically resolved masses in precursor (MS) and fragmentation (MS/MS) spectra were extracted using 3 nodes (without deconvolution, and with deconvolution using Xtract or MS2 Processor) in Proteome Discoverer (PD) software (v1.4, Thermo-Fisher Scientific). All extracted data were searched using Mascot (2.5.1; www.matrixscience.com) against the RefSeq2015 Mus musculus or RefSeq2015_mammals, each with an auto-concatenated decoy reversed database. The search criteria included: sample's species; trypsin as the enzyme, allowing one missed cleavage; cysteine carbamidomethylation as fixed modification; and methionine oxidation, and asparagine and glutamine deamidation as variable modifications. Proteome Discoverer uses only the peptide identifications with the highest Mascot score for the same peptide matched spectrum from the 3 different extract nodes and filtered the peptide identifications to 1% False Discovery Rate confidence based on the concatenated decoy database search.

RNA isolation and Reverse Transcription Polymerase Chain Reaction

50 mg of adult mouse (6-month old) heart tissue and human donor LV were treated with 1 mL of TRIZOL® Reagent (Thermo Fisher Scientific, USA) to obtain total RNA, which was reverse transcribed with the QuantiTect Reverse Transcription kit (Qiagen, Valencia, CA). 250 ng of cDNA were used in a PCR reaction with the GoTaq Green Master Mix (Promega, Madison, WI, USA) for amplification of the unique COOH-terminus of obscurin-B (amplicon size: 976 nts) or control GAPDH (amplicon size: 332 nts). PCR products were resolved in 1% agarose gels. The relevant primer sets are included in **Supplemental Table 1**.

Supplemental Figures

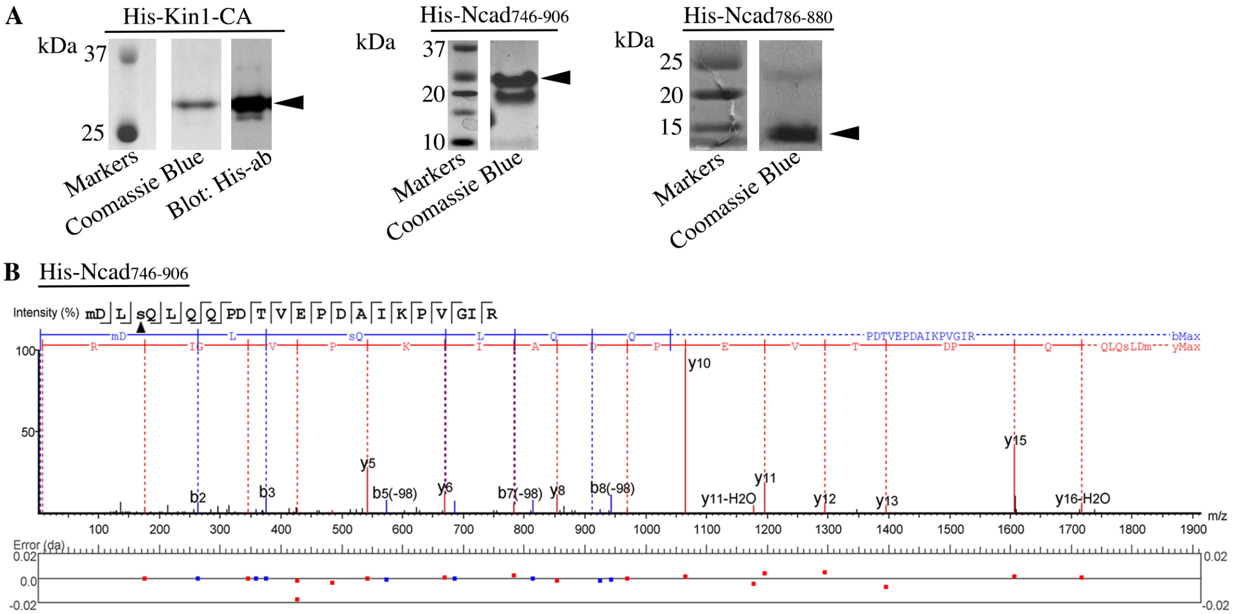

b	b-$H_2$0	b-NH_3	b (2+)	Seq	y	y-$H_2$0	y-NH_3	y (2+)
543.15	525.14	526.13	272.08	**s (+79.97)**	2271.14	2253.13	2254.11	1136.07

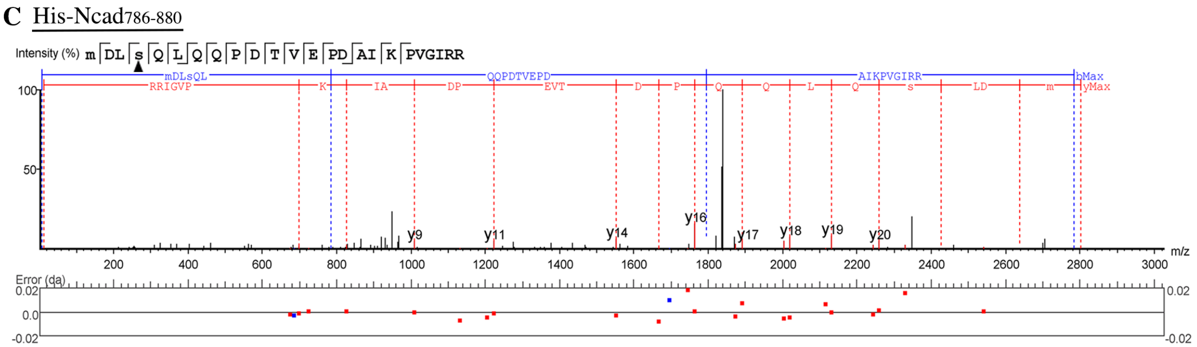

b	b-$H_2$0	b-NH_3	b (2+)	Seq	y	y-$H_2$0	y-NH_3	y (2+)
543.15	525.14	526.13	272.08	**s (+79.97)**	2427.24	2409.23	2410.21	1214.12

D

```
    Human N-cadherin: DEEGGGEEDQDYDLSQLQQPDTVEPDAIKPV
    Mouse N-cadherin: DEEGGGEEDQDYDLSQLQQPDTVEPDAIKPV
      Rat N-cadherin: DEEGGGEEDQDYDLSQLQQPDTVEPDAIKPV
   Bovine N-cadherin: DEEGGGEEDQDYDLSQLQQPDTVEPDAIKPV
  Chicken N-cadherin: DEEGGGEEDQDYDLSQLQQPDTVEPDAIKPV
Zebrafish N-cadherin: DEEGGGEEDQDYDLSQLQQPDTLEPDMIKPV
                      **********************:*** ****
```

```
 N-cadherin: DEEGGGEEDQD-YDLSQLQQPDTVEPDAIKPV
 E-cadherin: DEEGGGEEDQD-FDLSQLHRGLDARPEVT---
VE-cadherin: DEEGGGEMDTTSYDVSVLNSVRRGGAKPPRP-
 M-cadherin: DEQGGGEEDQDAYDISQLRHPTALSLPL-GPP
 R-cadherin: DEEGGGEEDQD-YDLSQLQQPEAMGHVPSKAP
             **:**** *    :*:* *.
```

Supplemental Figure 1

Supplemental Figure 1: Phosphoproteomics analysis of the obscurin-kin1/N-cadherin reaction. A. Baculovirus expressed His-Kin1-CA and bacterially expressed His-Ncad746-906 and His-Ncad786-880 were affinity-purified and evaluated by SDS-PAGE followed by Coomassie Blue staining before they were used in *in vitro* kinase assays; the identity of His-Kin1-CA was further

verified via immunoblotting using a His-antibody. **B-C:** MS/MS spectra of the N-cadherin peptides carrying phospho-Ser-788 (arrowhead) identified in *in vitro* kinase assays using His-Kin1-CA and either His-Ncad$_{746\text{-}906}$ **(B)** or His-Ncad$_{786\text{-}880}$ **(C)**. The indicated phospho-peptides were identified 1 and 6 times with a 100% probability as indicated by the lack of error following incubation of the His-Ncad$_{746\text{-}906}$ and His-Ncad$_{786\text{-}880}$ recombinant proteins, respectively, with His-Kin1-CA but not control baculovirus preparation. The ion values showing the addition of 79.97 Da to Ser-788 following incubation with His-Kin1-CA, which is indicative of the addition of a phosphate group are provided; n=2 independent mass spectrometry experiments. **D:** Multiple sequence alignment using Clustal Omega software indicated that Ser-788 is well conserved across species and within the cadherin family.

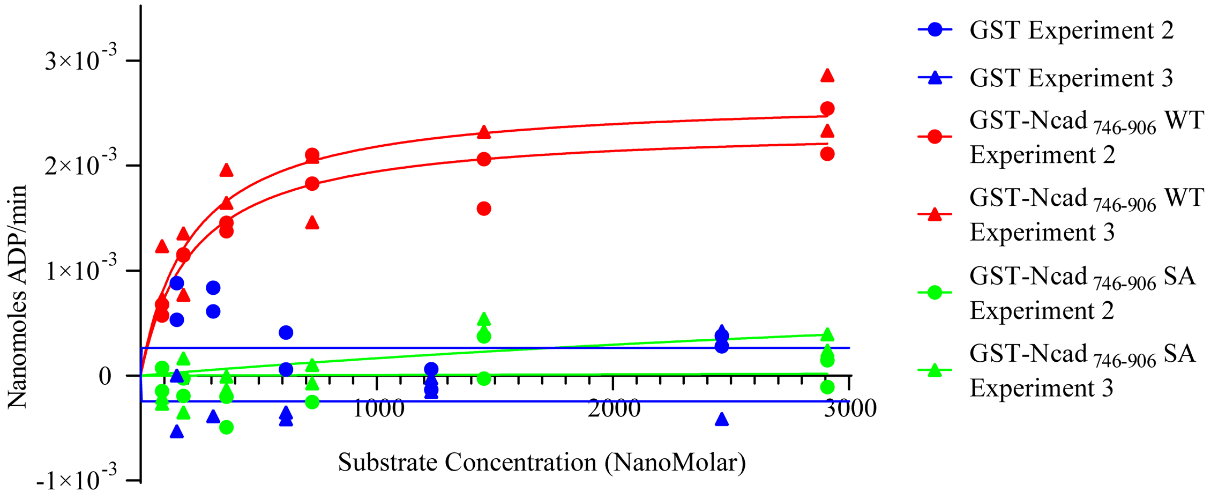

	GST-Ncad$_{746-906}$WT Experiment 2	GST-Ncad$_{746-906}$WT Experiment 3
Vmax (Nanomoles/min)	0.002377	0.002654
Km (Nanomolar)	222.9	217.3
Kcat (min^{-1})	4.57	5.10

Supplemental Figure 2

Supplemental Figure 2: Michaelis-Menten plots showing the nmoles of ADP produced per min at constant amount of His-Kin1-CA (26 nM) and ATP (100 μM) and varying substrate concentrations (90-2900 nM). The apparent Vmax, Km, and Kcat for GST-Ncad$_{746-906}$WT were calculated from two independent experiments performed in duplicate, yielding similar values to those shown in Fig. 3A. Use of GST and GST-Ncad$_{746-906}$SA did not produce any appreciable ADP formation.

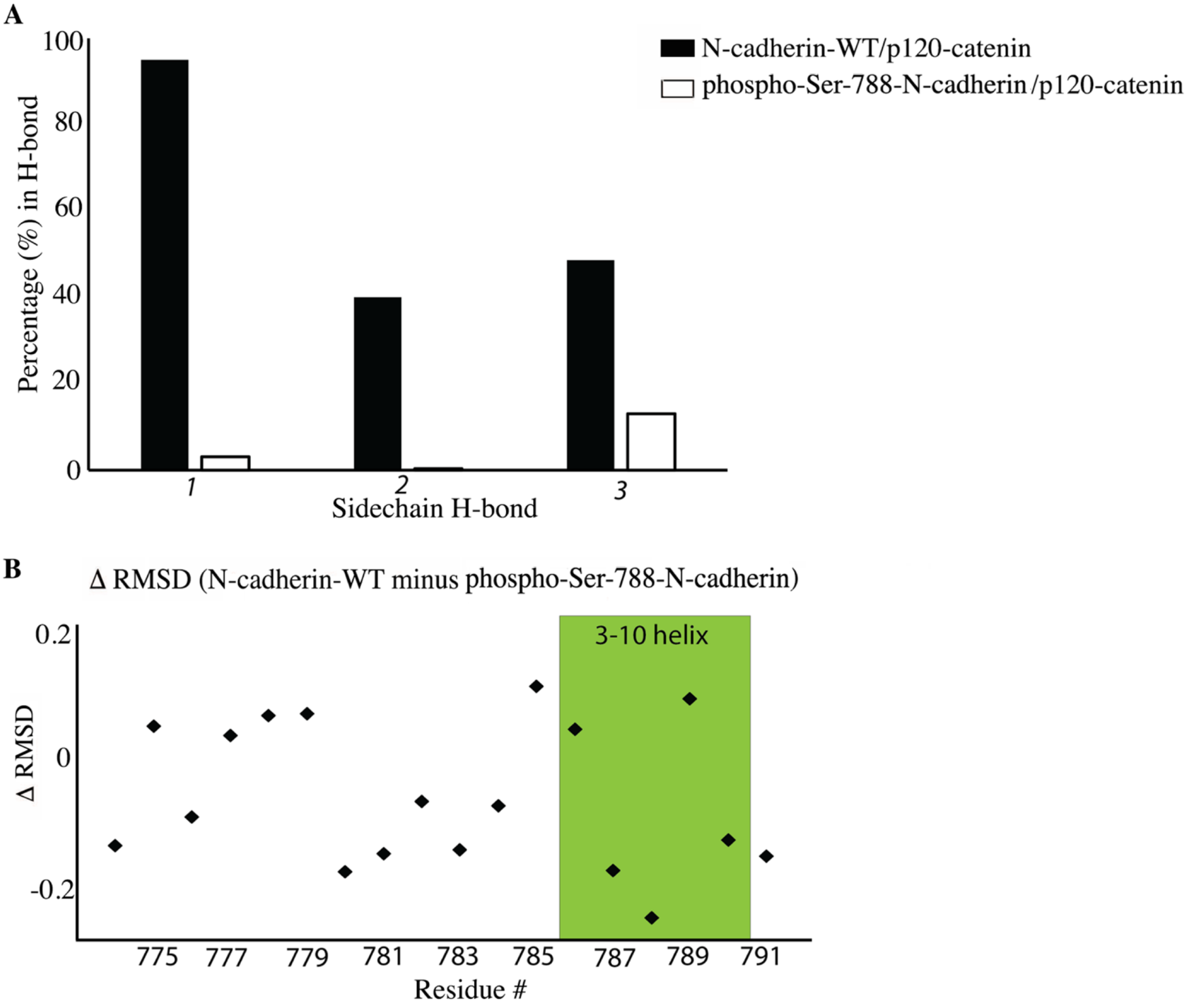

Supplemental Figure 3

Supplemental Figure 3: MD simulation of phospho-Ser-788 N-cadherin bound to p120-catenin. A: In a 140 ns MD simulation of p120-catenin bound N-cadherin D774-H791 model, permutated to include phosphorylated Ser-788, the 3-10 helix that supports hydrophobic interactions with p120-catenin loses multiple stabilizing hydrogen bonds. This closely mirrors the phosphomimic N-cadherin-SE/p120-catenin data presented in Fig. 6B. This loss of hydrogen bonds partially destabilizes the cadherin helix, resulting in modestly increased motion surrounding the p120-catenein hydrophobic patch when compared to WT N-cadherin. This is evidenced by a

decreased ΔRMSD in the residues that interact closely with the corresponding p120-catenin hydrophobic patch (Leu-787, Ser-788, and Leu-790). Note that there is more residue-by-residue variability between WT and the phospho-Ser-N-cadherin in this simulation than in the N-cadherin-SE simulation presented in Figure 6.

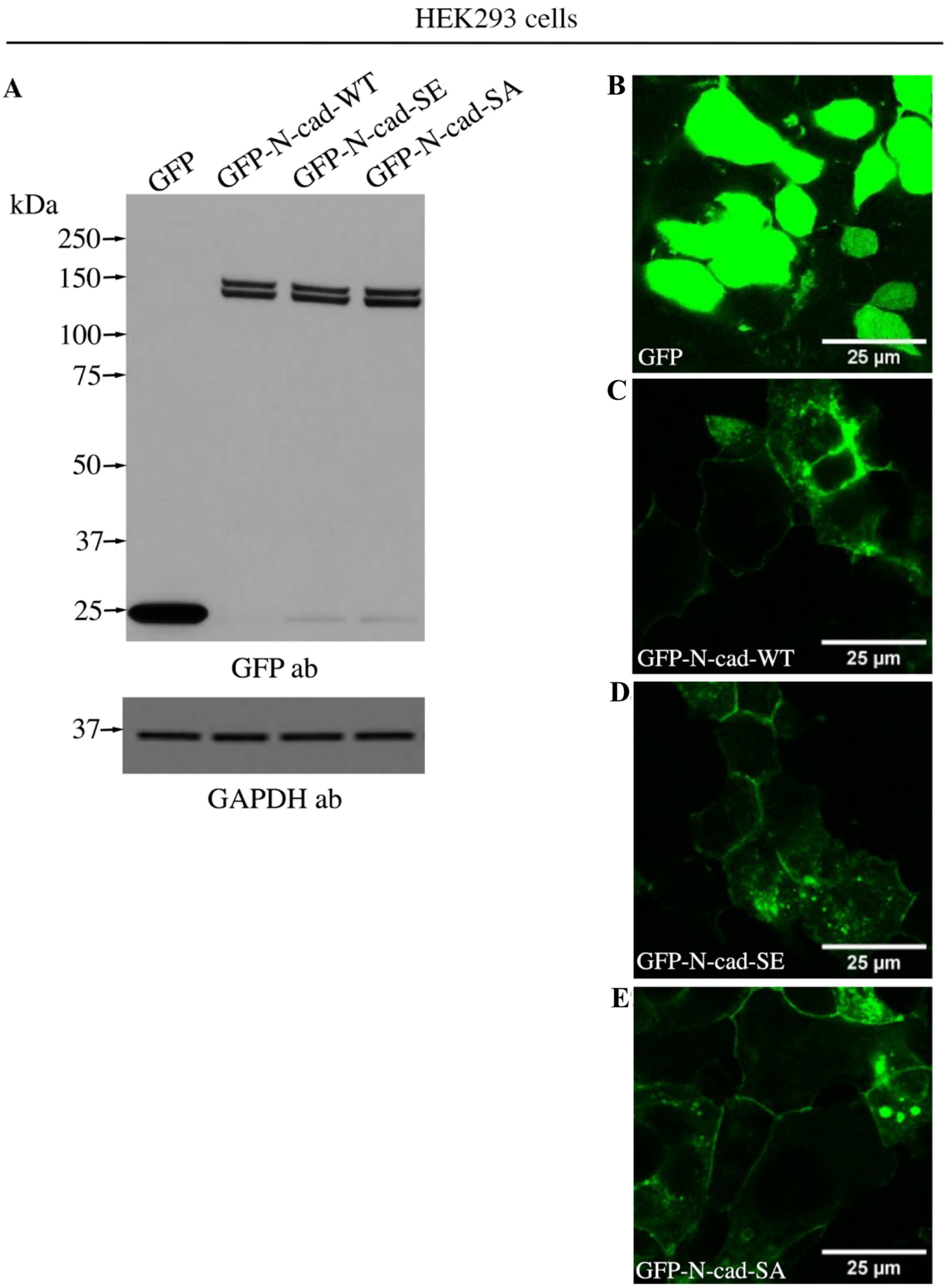

Supplemental Figure 4

Supplemental Figure 4: Expression and membrane localization of exogenous wild type, phosphomimic and phosphoablated N-cadherin in HEK293 cells. A. Immunoblotting assays probed with GFP-antibody showing similar expression levels of exogenous GFP-tagged full length

WT (GFP-N-cad-WT), phosphomimic SE (GFP-N-cad-SE), and phosphoablated SA (GFP-N-cad-SA) N-cadherin proteins as well as control GFP in HEK293 cells; GAPDH was used as loading control. Of note, a slightly smaller degradation product was detected in all three exogenous N-cadherin variants that is still recognized by the GFP-antibody. **B-E.** Representative images of GFP fluorescence obtained under confocal optics indicating that exogenous GFP-tagged full length WT (GFP-N-cad-WT), phosphomimic SE (GFP-N-cad-SE) and phosphoablated SA (GFP-N-cad-SA) N-cadherin proteins target to the cell membrane at sites of cell-cell contact, while control GFP protein occupies the entire cell.

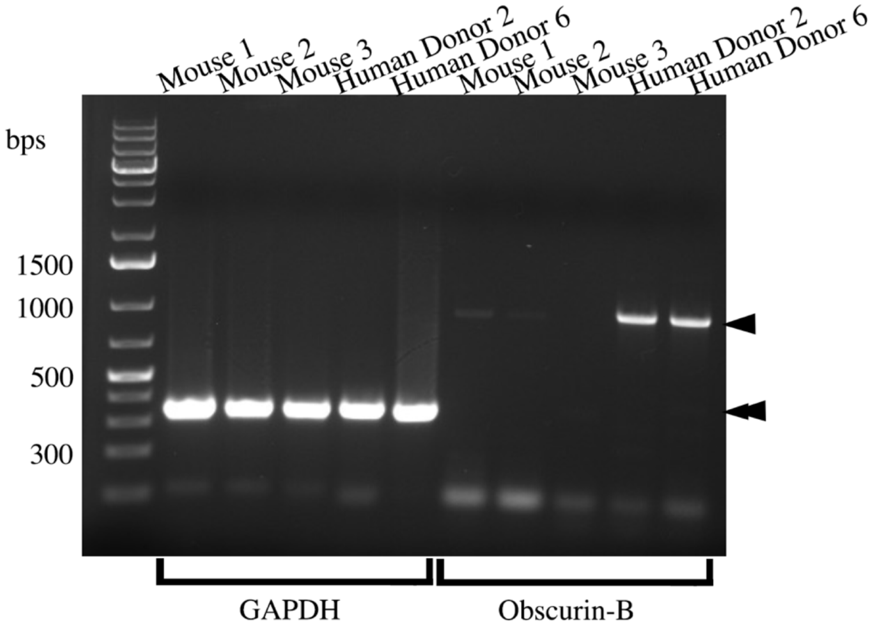

Supplemental Figure 5

Supplemental Figure 5: Expression of obscurin-B transcripts in mouse and human myocardia. RT-PCR analysis of adult (6-month old) mouse myocardia and human donor 2 and 6 left ventricles using primers within obscurin-B's kinase linker region (7) and kin1 **(Supplemental Table 1)**. Obscurin-B amplicons (~980 bps) were readily detected in human donor samples, but not in mouse myocardia. Amplification of GAPDH transcripts served as internal control in parallel reactions.

References

1. Hu LY, and Kontrogianni-Konstantopoulos A. The kinase domains of obscurin interact with intercellular adhesion proteins. *FASEB J.* 2013;27(5):2001-12.
2. Nechiporuk T, Fernandez TE, and Vasioukhin V. Failure of epithelial tube maintenance causes hydrocephalus and renal cysts in Dlg5-/- mice. *Dev Cell.* 2007;13(3):338-50.
3. Ackermann MA, King B, Lieberman NAP, Bobbili PJ, Rudloff M, Berndsen CE, et al. Novel obscurins mediate cardiomyocyte adhesion and size via the PI3K/AKT/mTOR signaling pathway. *J Mol Cell Cardiol.* 2017;111:27-39.
4. Geisler SB, Green KJ, Isom LL, Meshinchi S, Martens JR, Delmar M, et al. Ordered assembly of the adhesive and electrochemical connections within newly formed intercalated disks in primary cultures of adult rat cardiomyocytes. *J Biomed Biotechnol.* 2010;2010:624719.
5. Kizana E, Chang CY, Cingolani E, Ramirez-Correa GA, Sekar RB, Abraham MR, et al. Gene transfer of connexin43 mutants attenuates coupling in cardiomyocytes: novel basis for modulation of cardiac conduction by gene therapy. *Circ Res.* 2007;100(11):1597-604.
6. Shevchenko A, Wilm M, Vorm O, and Mann M. Mass spectrometric sequencing of proteins silver-stained polyacrylamide gels. *Anal Chem.* 1996;68(5):850-8.
7. Fukuzawa A, Idowu S, and Gautel M. Complete human gene structure of obscurin: implications for isoform generation by differential splicing. *J Muscle Res Cell Motil.* 2005;26(6-8):427-34.

Supplemental Table 1

Primer	Sequence
Recombinant Protein Production (Vector)	
m-Ncad$_{CYT}$ (aa 746-906) sense (pGEX4T1)	5'-ACGTGGATCCGACTTGAGCCAGCTCCAGCAACC-3'
m-Ncad$_{CYT}$ (aa 746-906) antisense (pGEX4T1)	5'-ACGTCTCGAGTCACCCACCGCTACTGGAGGAGTTGA-3'
m-Ncad$_{CYT}$ (aa 746-906) sense (pET21a)	5'-ACGTCATATGAAACGGCGGGATAAAGAGCGCCA-3'
m-Ncad$_{CYT}$ (aa 746-906) antisense (pET21a)	5'-TGCACTCGAGGTCGTCACCACCGCCGTACATG-3'
m-Ncad$_{CYT}$-short (aa 786-880) sense (pET21a)	5'-ACGTCATATGGACTTGAGCCAGCTCCAGCAACC-3'
m-Ncad$_{CYT}$-short (aa 786-880) antisense (pET21a)	5'-TGCACTCGAGCCCACCGCTACTGGAGGAGTTG-3'
m-p120 (aa 311-747) sense (pMAL-c2E)	5'-ACTGAAGGTACCGGAATTCATGCCCTCTG-3'
m-p120 (aa 311-747) antisense (pMAL-c2E)	5'-ACTGAAGCTTCTAAATTAACTCTTTGTT-3'
Mutagenesis	
m-Ncad-SE sense	5'-CAGGACTATGACTTG*GAG*CAGCTCCAGCAACC-3'
m-Ncad-SE antisense	5'-GGTTGCTGGAGCTG*CTC*CAAGTCATAGTCCTG-3'
m-Ncad-SA sense	5'-CAGGACTATGACTTG*GCC*CAGCTCCAGCAACC-3'
m-Ncad-SA antisense	5'-GGTTGCTGGAGCTG*GGC*CAAGTCATAGTCCTG-3'
Reverse Transcription Polymerase Chain Reaction	
m/h-Obsc-Kinase Linker sense	5'-ACTGGAGACCACACAGAGGACC-3'
m/h-Obsc-Kin1 antisense	5'-ACTGCAAATGGGGATGAGCAGGT-3'
m/h-GAPDH sense	5'-ACTGAAGGCTGTGGGCAAGG-3'
m/h-GAPDH antisense	5'-ACTGTGAGGTCCACCACCCT-3'

m: mouse; h: human; CYT: cytoplasmic; SE: phosphomimic containing Glu-788; SA: phosphoablated containing Ala-788

Figure 1 A-C:

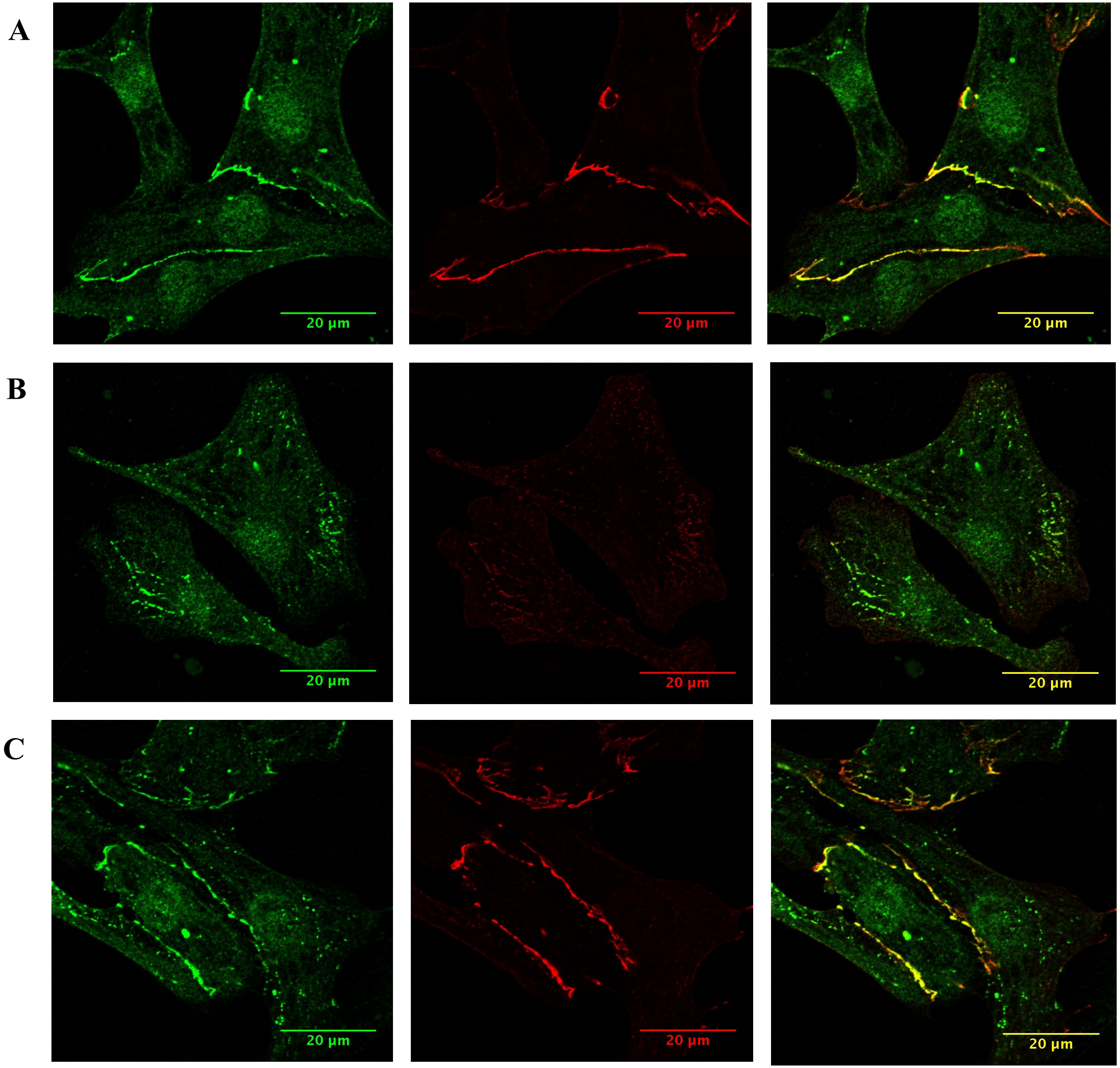

Figure 1 D-F:

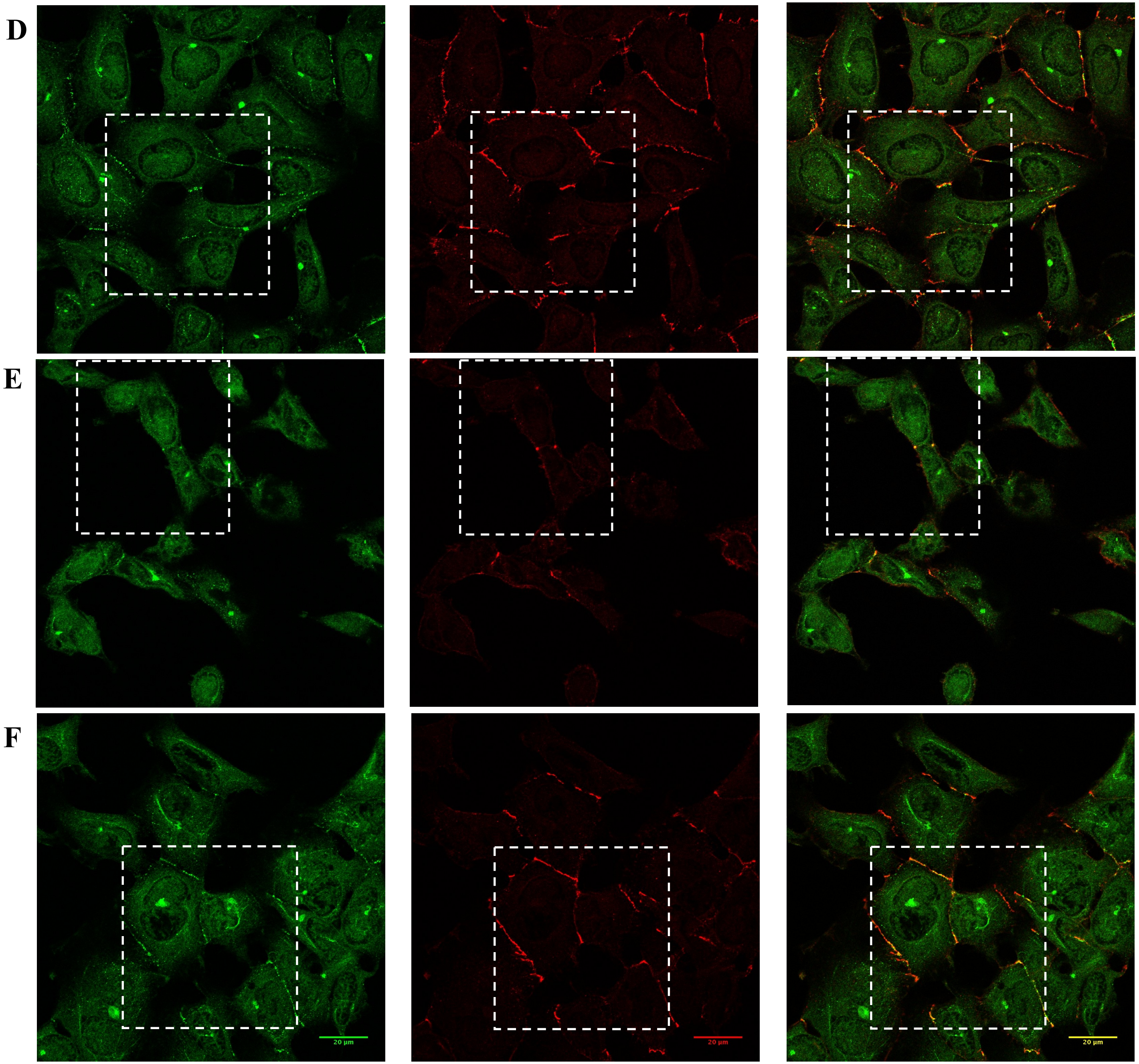

Note: Areas marked with white dashed boxes are shown in Fig. 1 D-F.

Figure 1 G:

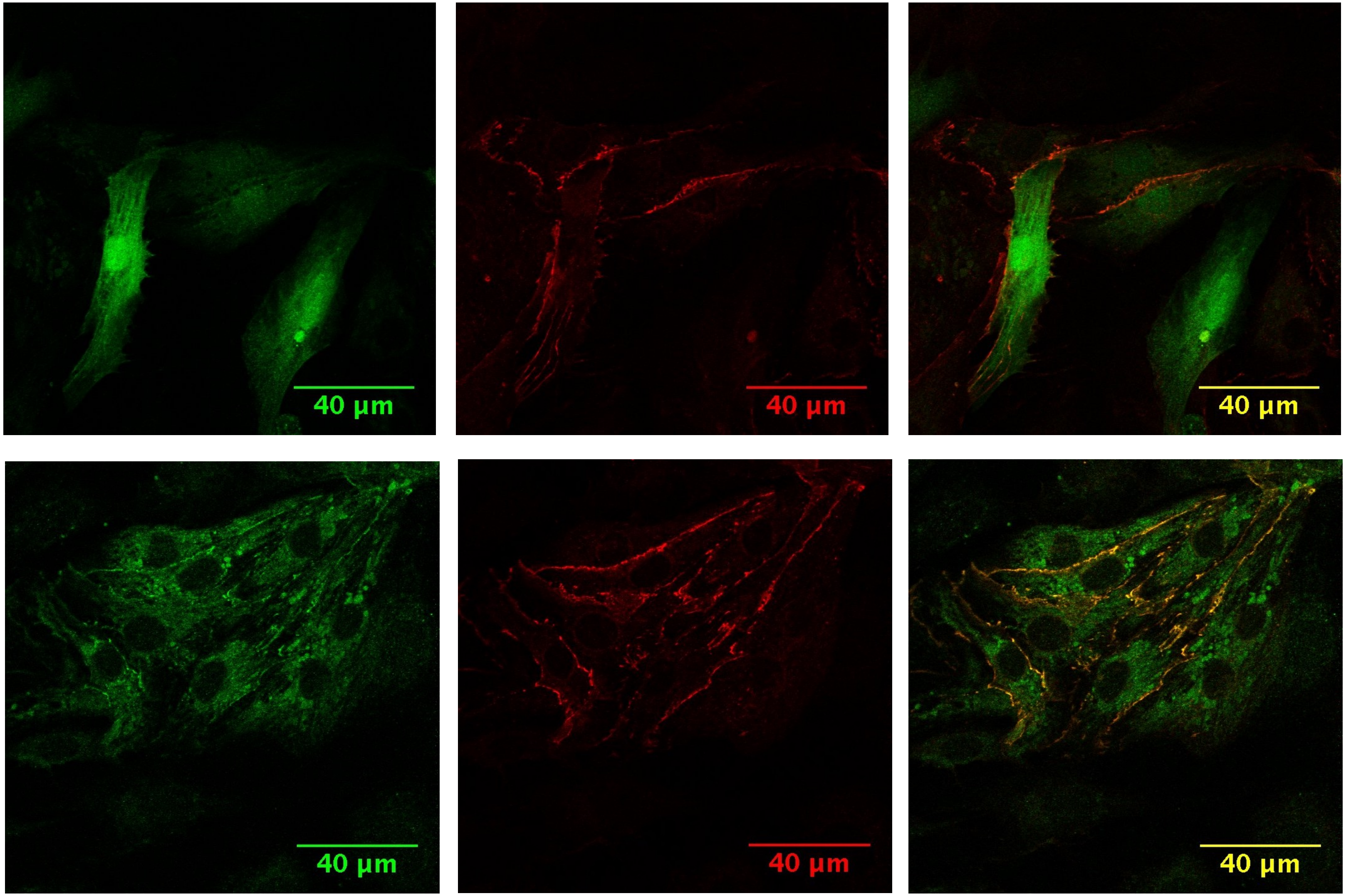

Figure 1H: **#1, #2, #3: Replicate experiments**

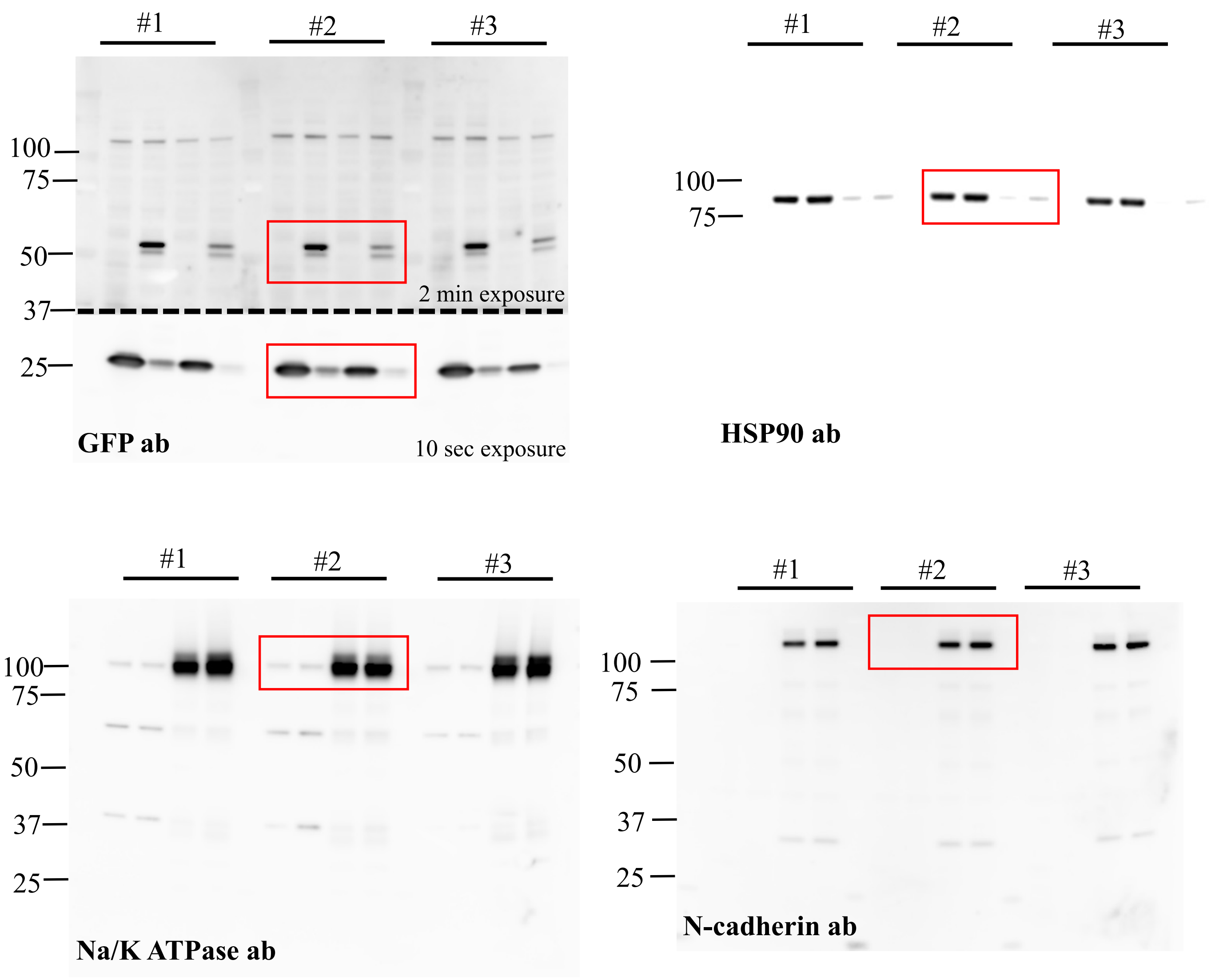

Note: These are the raw images obtained from the PhosphoImager; for consistency of presentation, blots in Fig. 1H are presented in grayscale mode; red boxes indicate the lanes/bands used in Fig. 1H .

Figure 2A

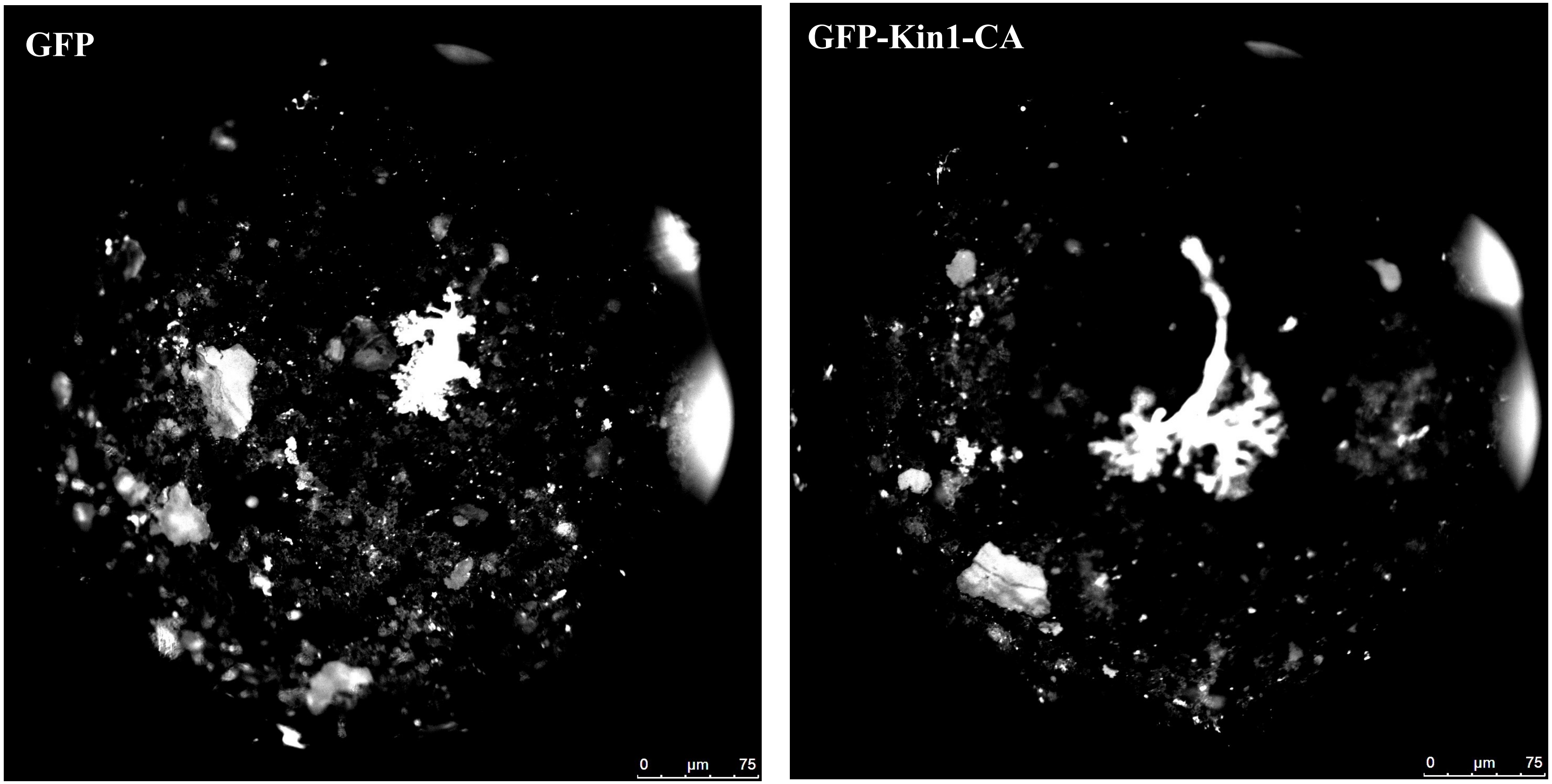

Figure 3B

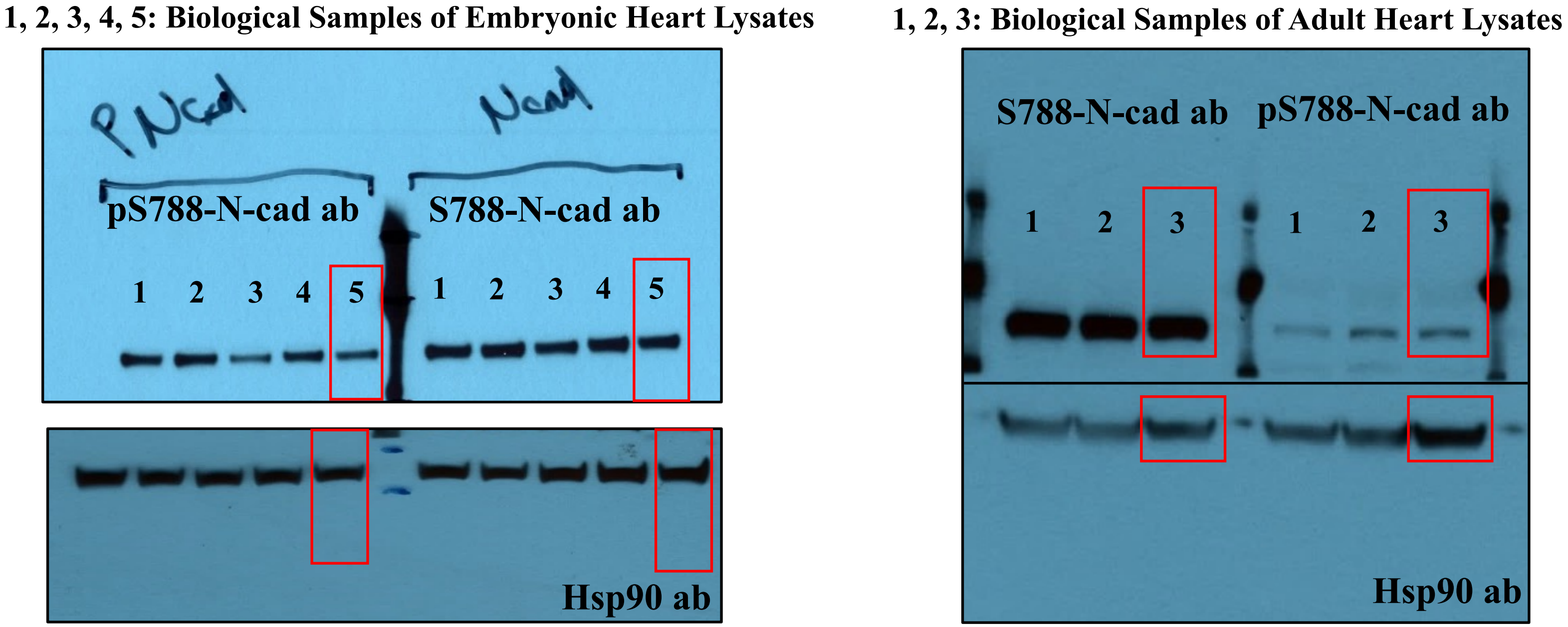

Figure 3E

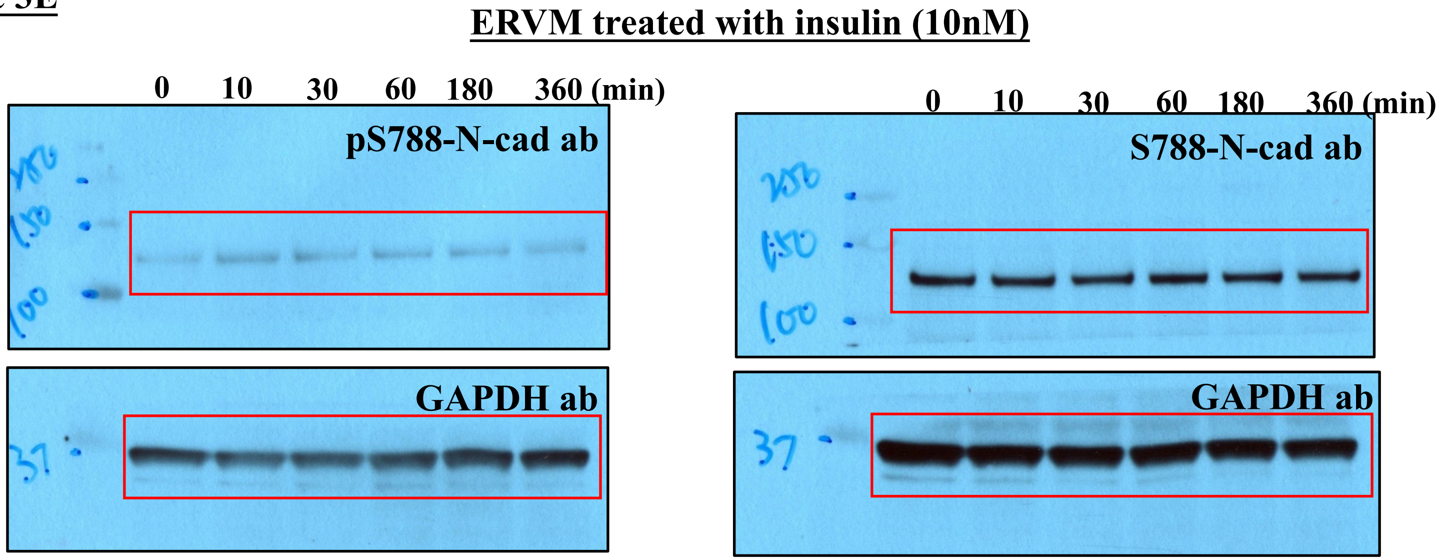

Note: Blots in Fig. 3B and E are presented in grayscale mode; red boxes indicate the lanes/bands used in Fig. 3B and E.

Figure 3C

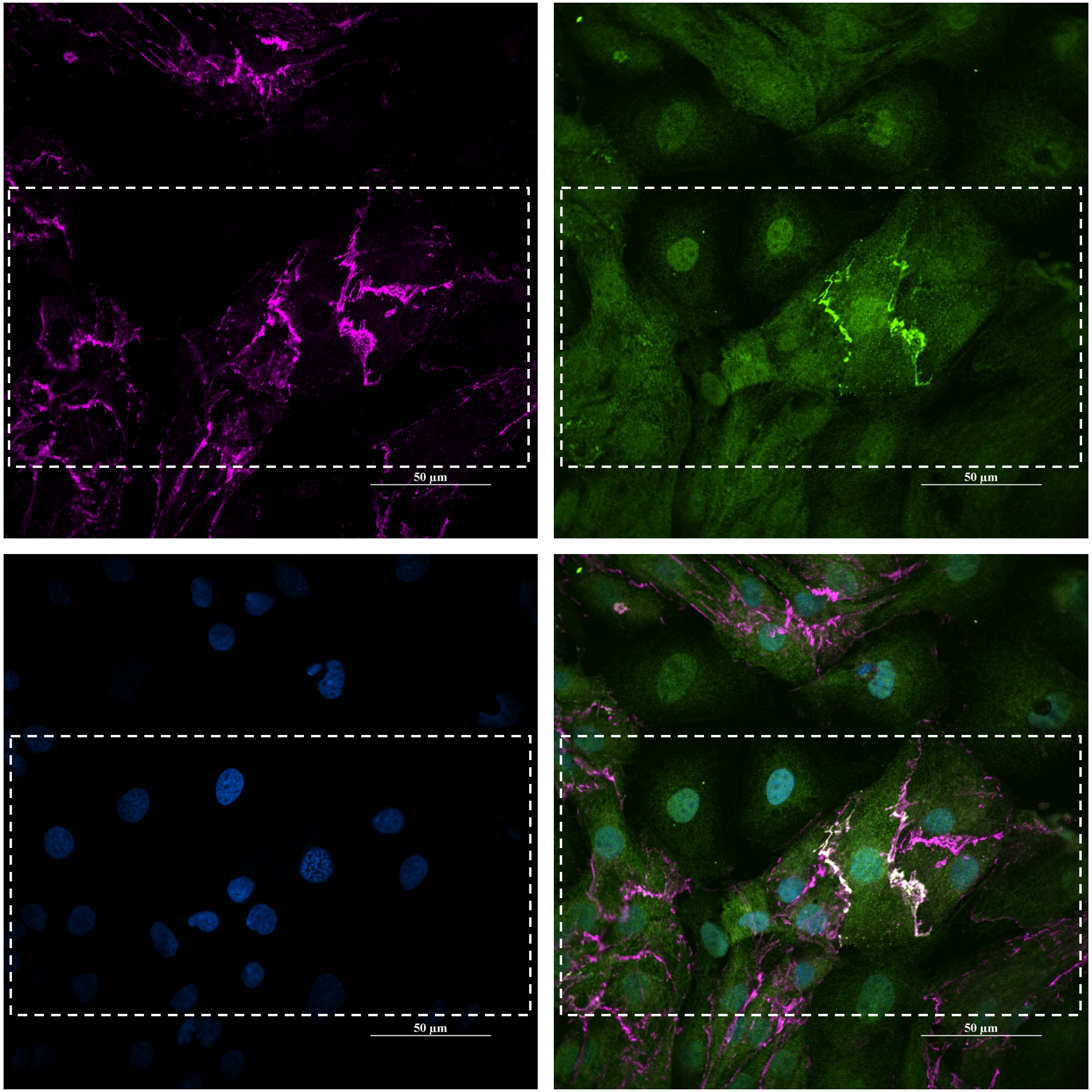

Note: Areas marked with white dashed boxes are shown in Fig. 3C.

Figure 3D

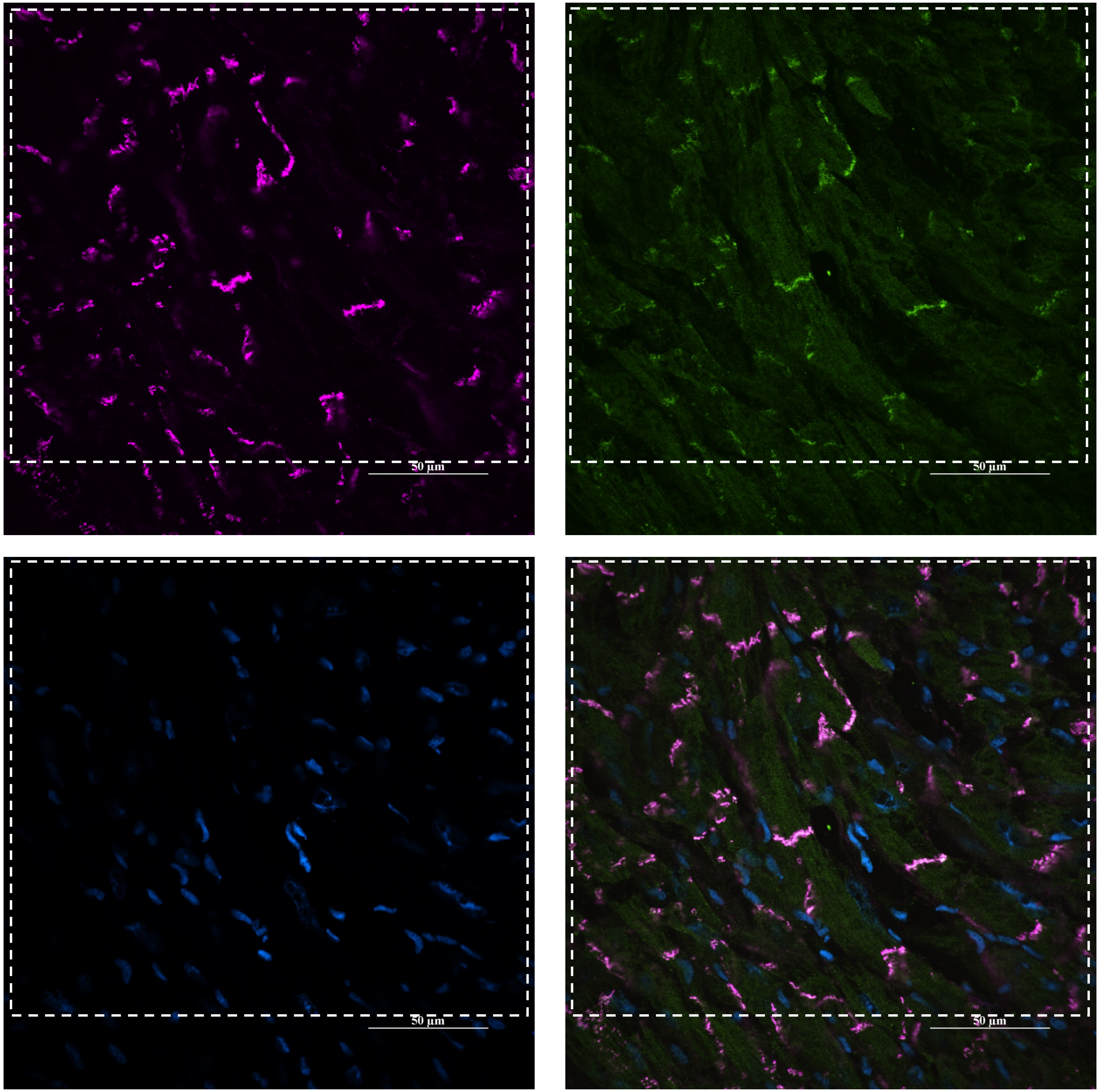

Note: Areas marked with white dashed boxes are shown in Fig. 3D.

Figure 4 A

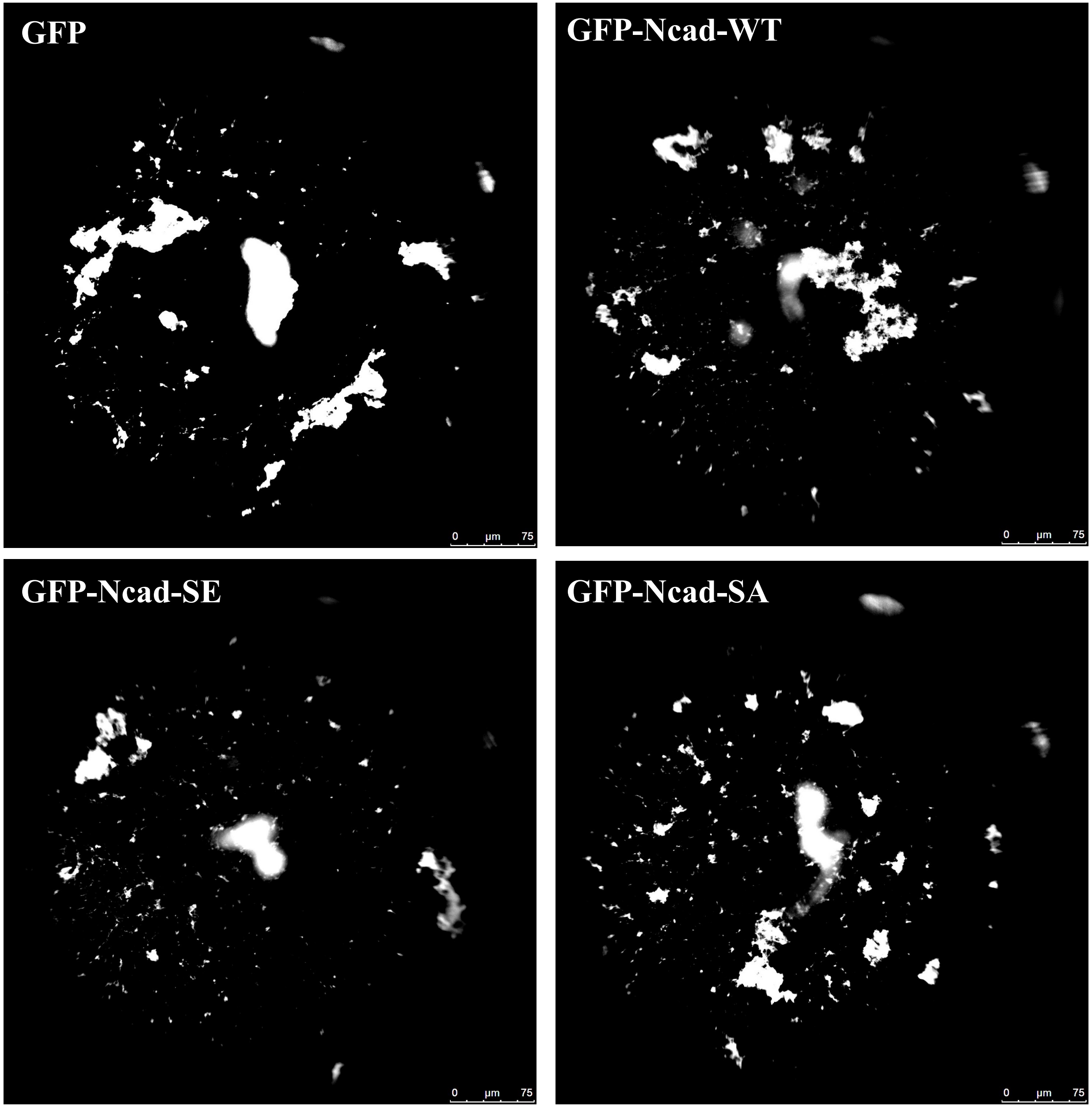
GFP
GFP-Ncad-WT
GFP-Ncad-SE
GFP-Ncad-SA
0 μm 75
0 μm 75
0 μm 75
0 μm 75

Figure 5 A-B

A

1: GST - **2:** GST-Ncad$_{746\text{-}906}$-WT - **3:** GST-Ncad$_{746\text{-}906}$-SE - **4:** GST-Ncad$_{746\text{-}906}$-SA - **5:** Heart lysates

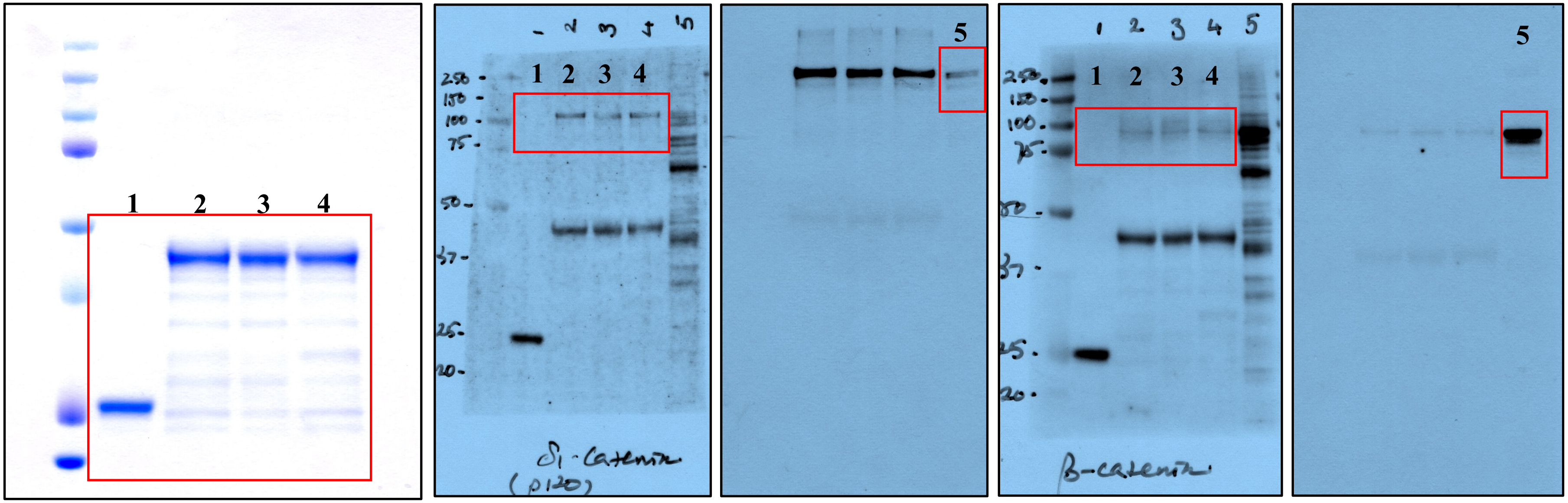

Coomassie Blue **p120 catenin ab** **β-catenin ab**

B

1: GST - **2:** GST-Ncad$_{746\text{-}906}$-WT - **3:** GST-Ncad$_{746\text{-}906}$-SE - **4:** MBP-p120$_{311\text{-}747}$

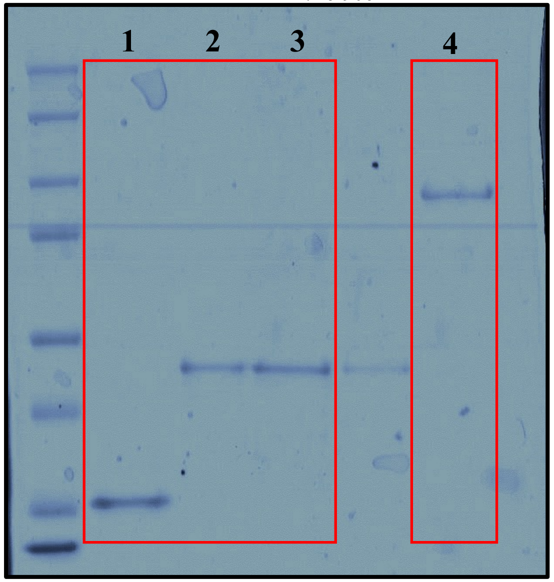

Coomassie Blue

Note: Gels and blots in Fig. 5A-B are presented in grayscale mode; red boxes indicate the lanes/bands used in Fig. 5 A-B.

<u>**Figure 6E**</u>

A, B, C: Replicate experiments

1: GFP - **2:** GST-Ncad-WT - **3:** GST-Ncad-SE - **4:** GST-Ncad-SA

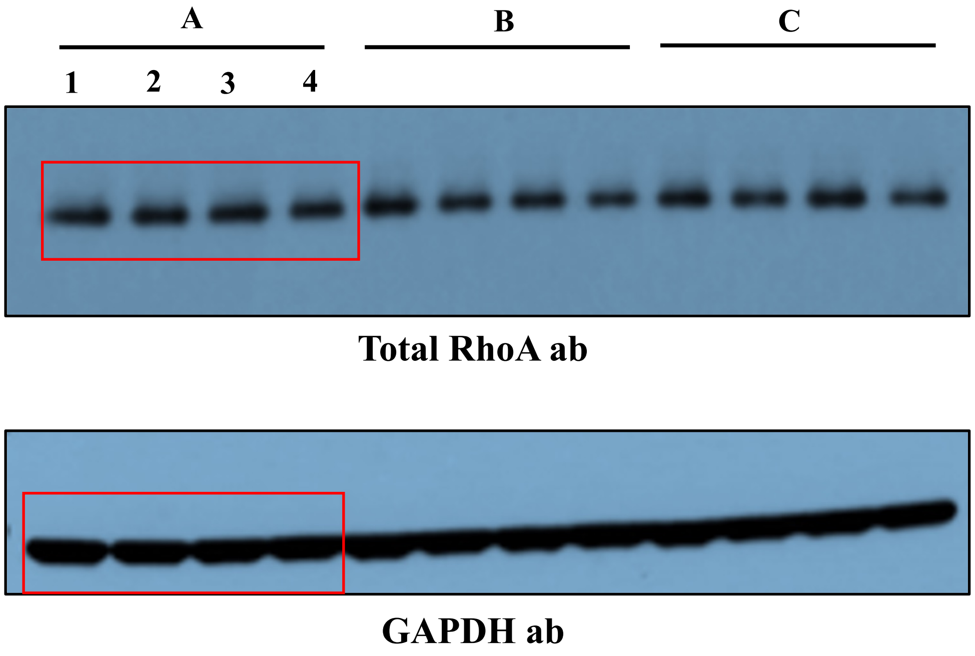

Note: Blots in Fig. 6E are presented in grayscale mode; red boxes indicate the lanes/bands used in Fig. 6E.

Figure 7

Obsc-kin1 ab blots: Samples 1-6: Donors-Samples 7-15: DCM

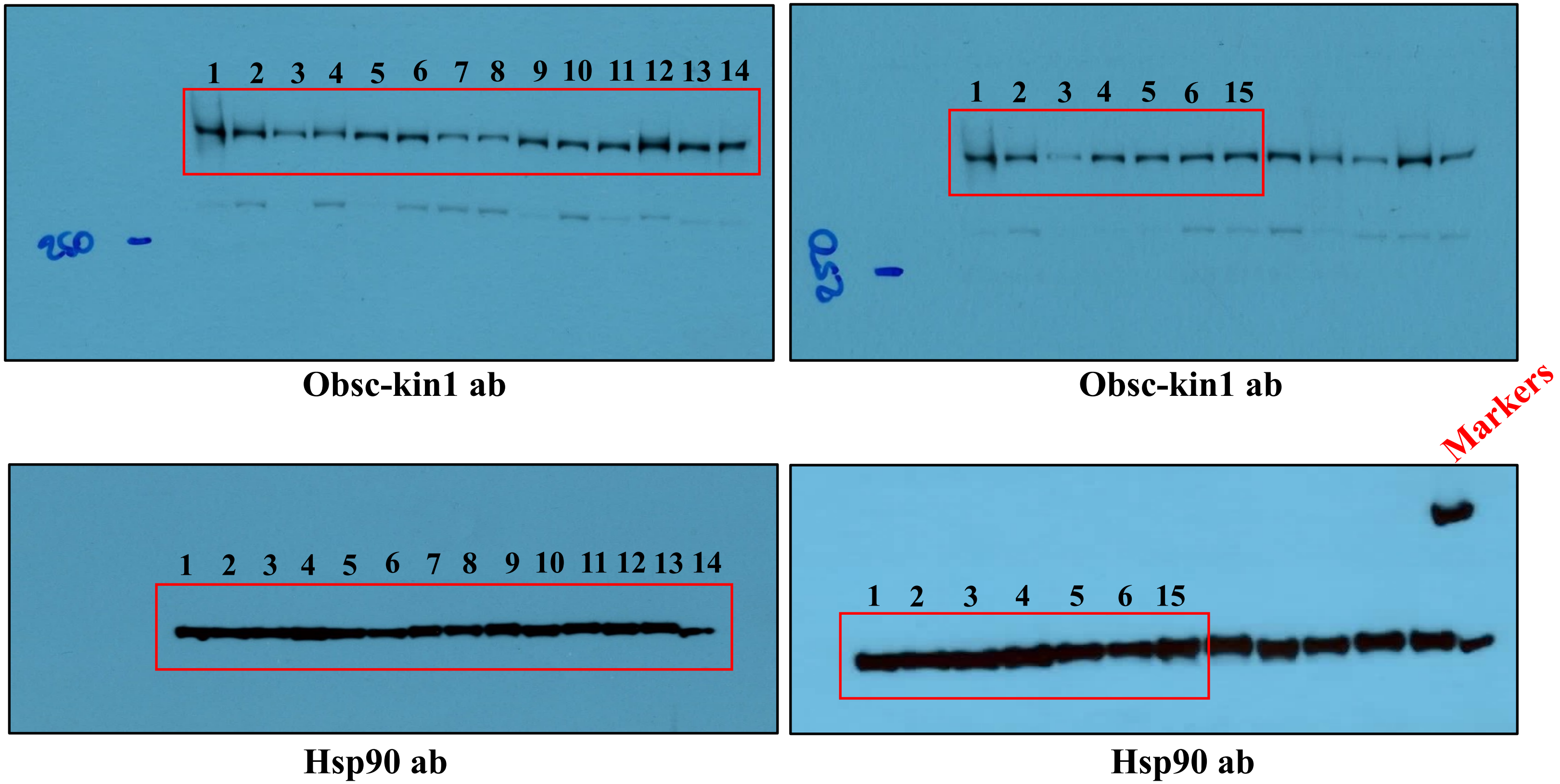

Note: Blots in Fig. 7 are presented in grayscale mode; red boxes indicate the lanes/bands used in Fig. 7.

Figure 7

pNcad/Ncad ab blots: Samples 1-6: Donors-Samples 7-15: DCM

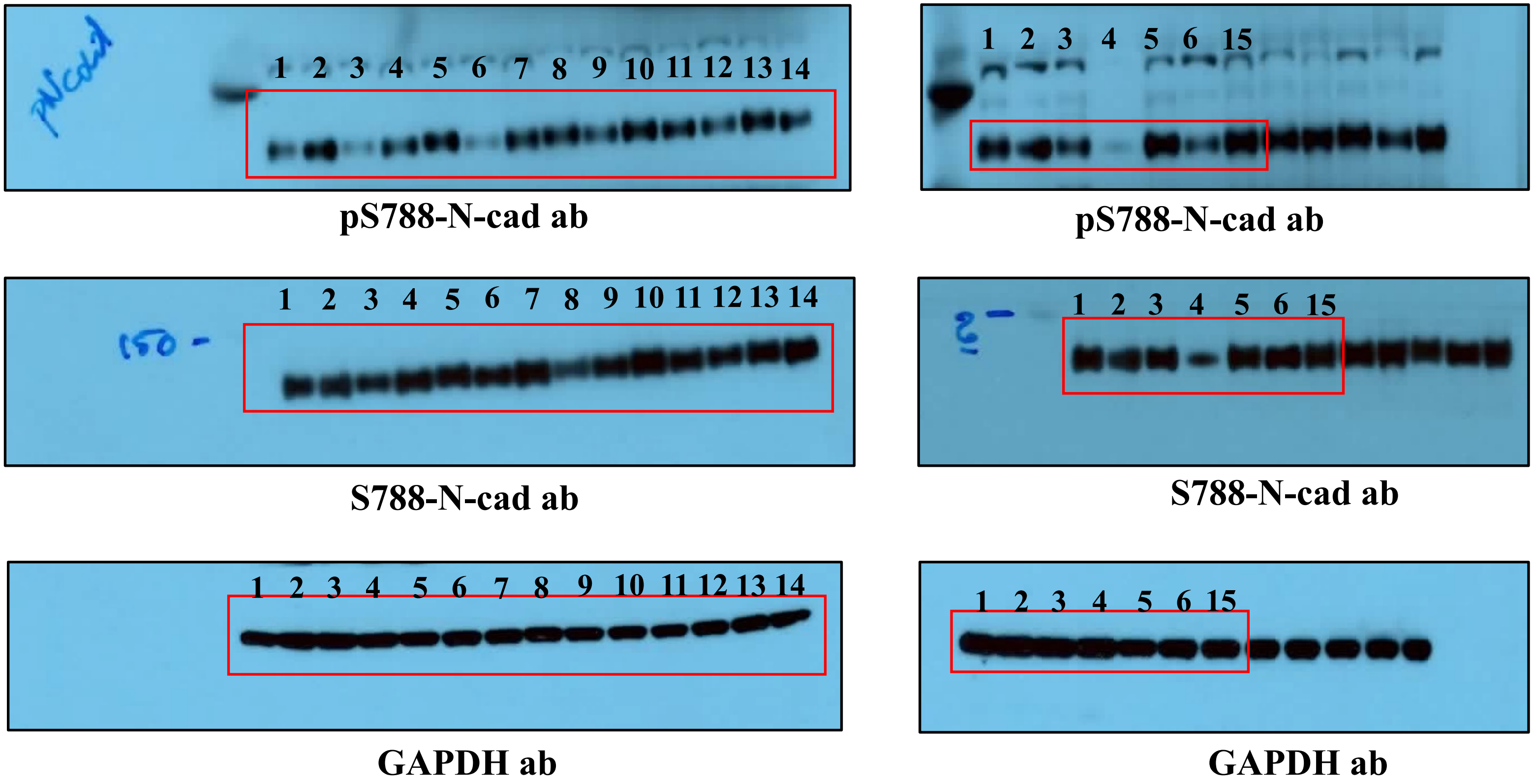

Note-1: Blots in Fig. 7 are presented in grayscale mode; red boxes indicate the lanes/bands used in Fig. 7.

Supplemental Figure 1A

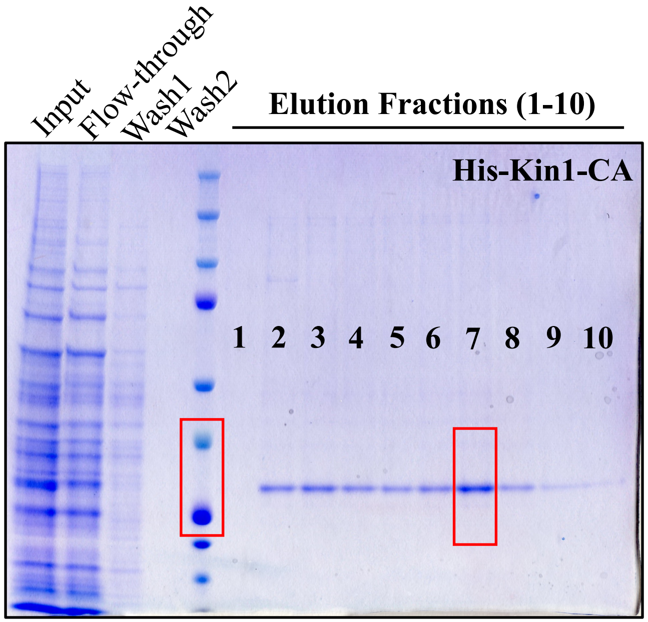

Coomassie Blue

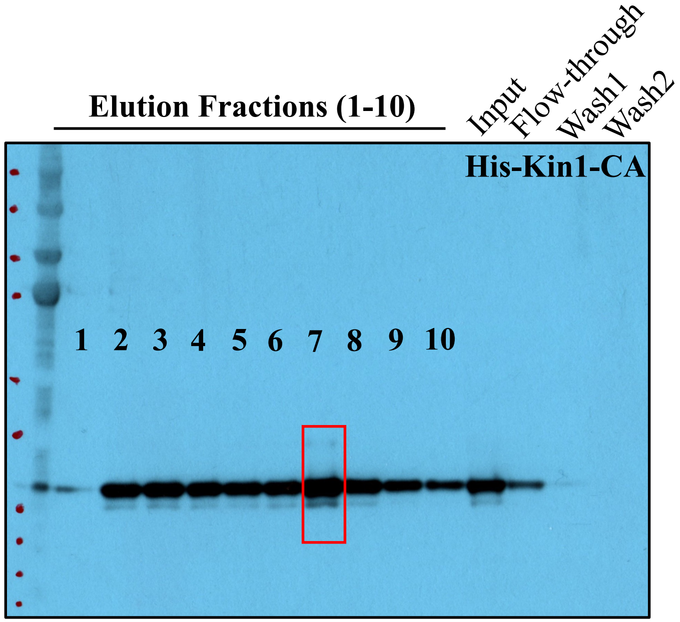

Blot: His-ab

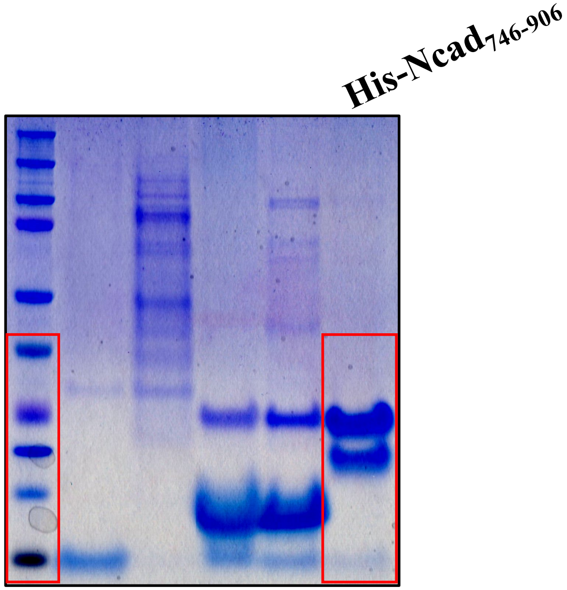

Coomassie Blue

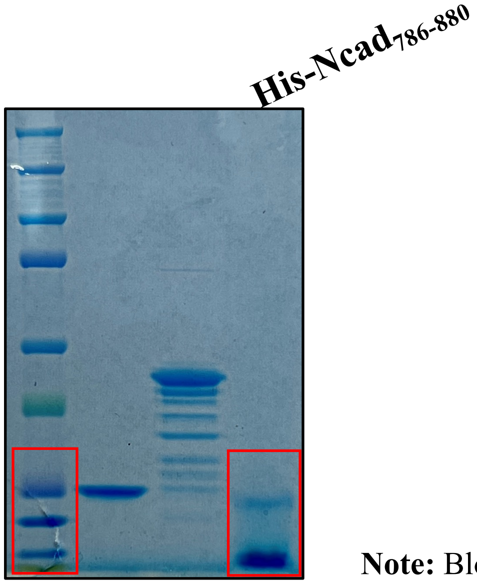

Coomassie Blue

Note: Blots in Fig. S1A are presented in grayscale mode; red boxes indicate the lanes/bands used in the figure.

Supplemental Figure 4A

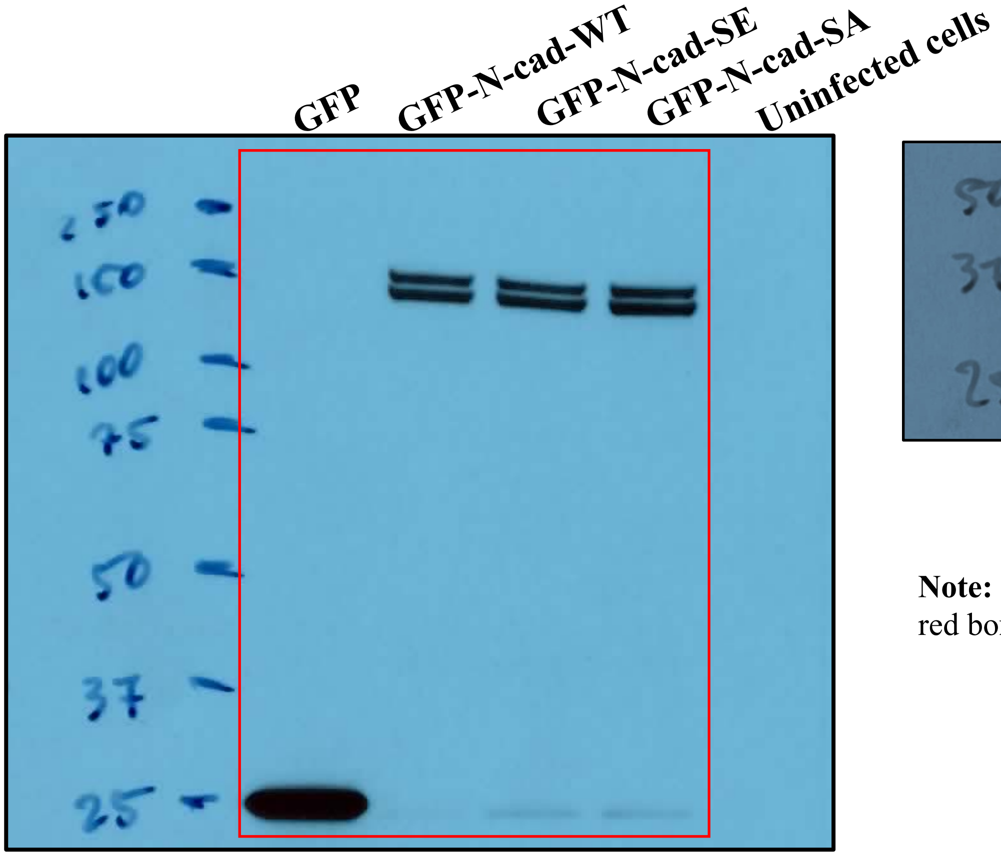

GFP ab

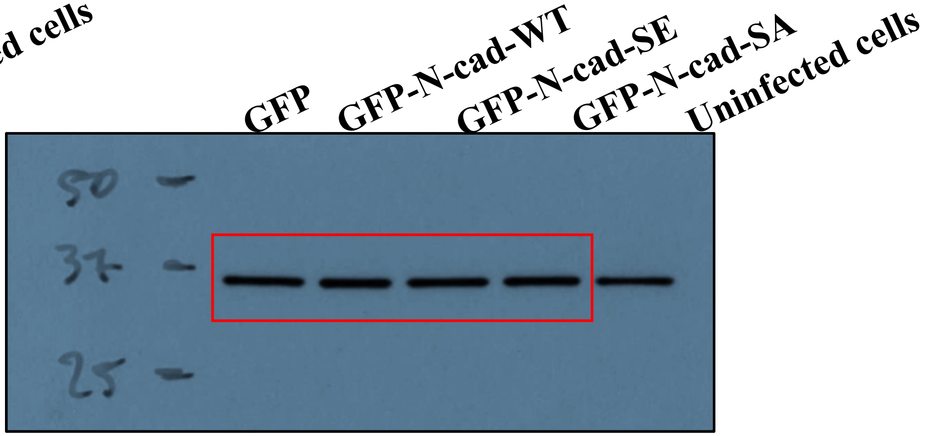

GAPDH ab

Note: Blots in Fig. S2A are presented in grayscale mode; red boxes indicate the lanes/bands used in the figure.

Supplemental Figure 4 B-E

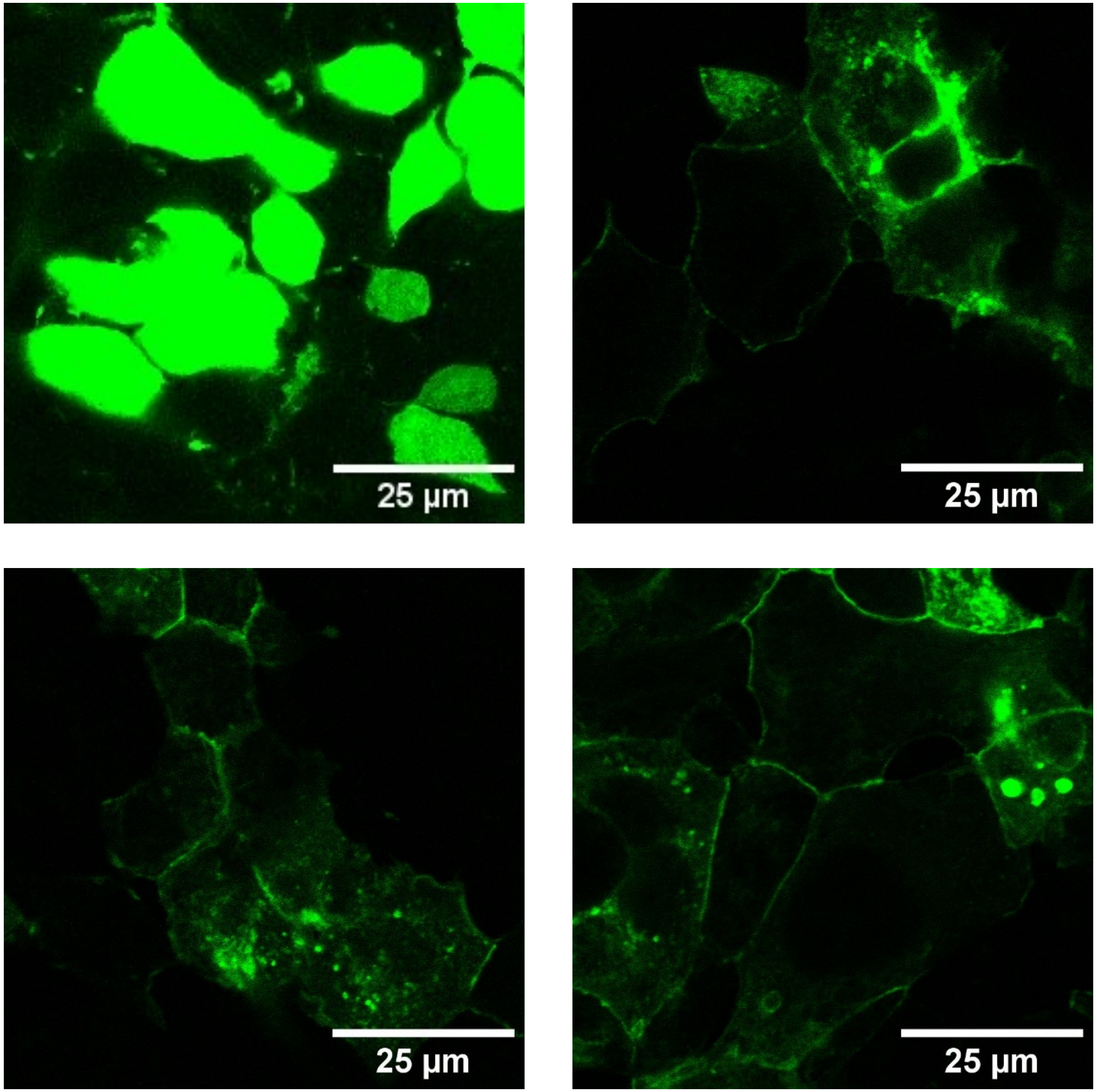

Supplemental Figure 5

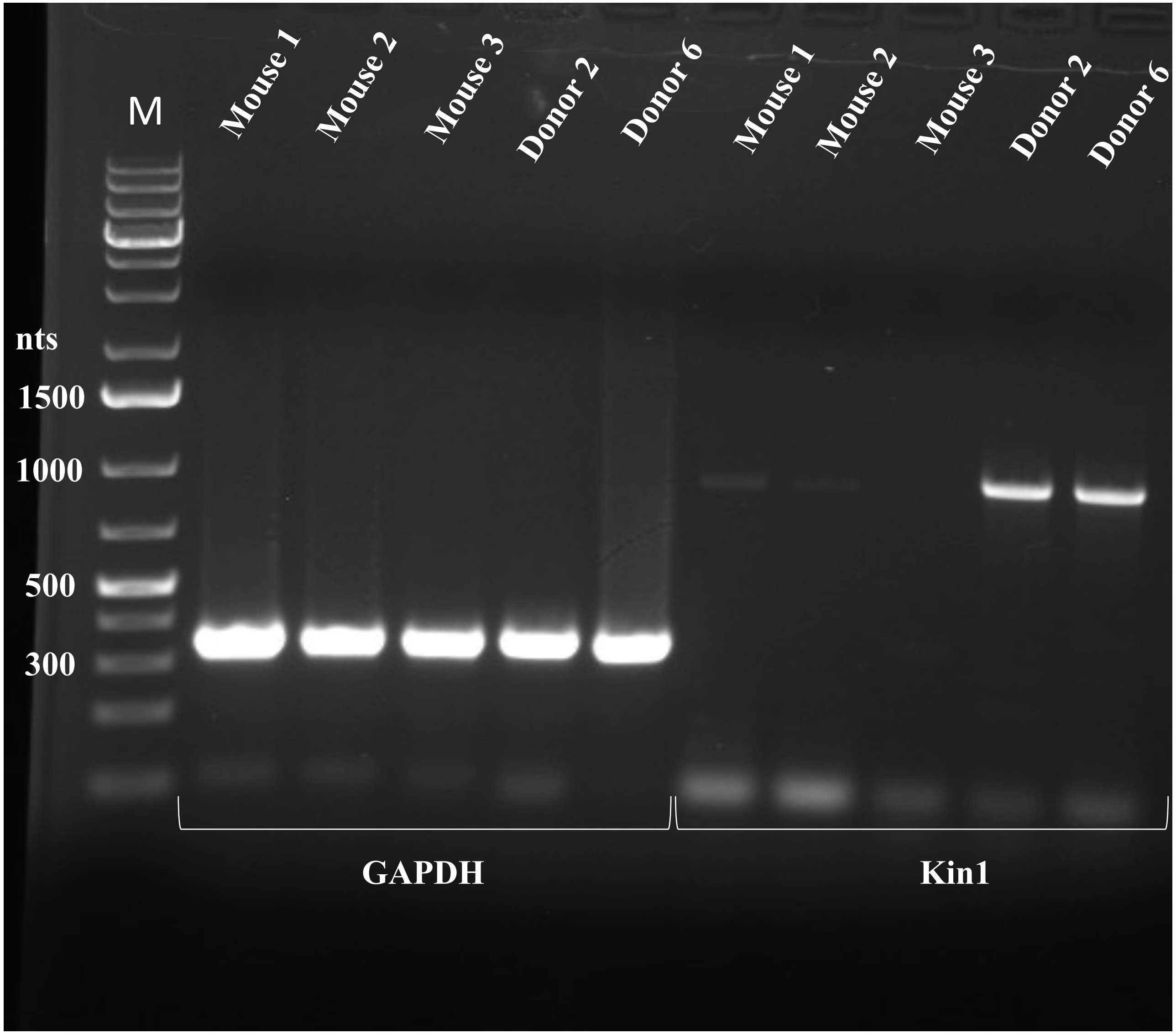

Supplement: Supplemental data [file jciinsight-9-162178-s075.pdf]
